# An investigation of the observed, but counter-intuitive, stereoselectivity noted during chiral amine synthesis via *N*-chiral-ketimines

**DOI:** 10.3762/bjoc.9.247

**Published:** 2013-10-15

**Authors:** Thomas C Nugent, Richard Vaughan Williams, Andrei Dragan, Alejandro Alvarado Méndez, Andrei V Iosub

**Affiliations:** 1 Department of Chemistry, School of Engineering and Science, Jacobs University Bremen, Campus Ring 1, 28759 Bremen, Germany; 2 Department of Chemistry, University of Idaho, PO Box 442343, Moscow, ID 83844-2343, USA

**Keywords:** chiral amines, *cis/trans* isomerization, imine isomerization, imine reduction, reductive amination

## Abstract

The default explanation for good to high diastereomeric excess when reducing *N*-chiral imines possessing only mediocre *cis*/*trans*-imine ratios (>15% *cis*-imine) has invariably been in situ *cis*-to-*trans* isomerization before reduction; but until now no study unequivocally supported this conclusion. The present study co-examines an alternative hypothesis, namely that some classes of *cis*-imines may hold conformations that erode the inherent facial bias of the chiral auxiliary, providing more of the *trans*-imine reduction product than would otherwise be expected. The ensuing experimental and computational (DFT) results favor the former, pre-existing, explanation.

## Introduction

A class of chiral compounds drawing ever more attention is α-chiral amines (chiral amines). Amines are known to be potent pharmacophores, and medicinal chemists have further leveraged their beneficial properties by using chiral versions to enhance protein binding affinities. Furthermore, chiral amines continue to find expanded roles: in organocatalysis [1–4], as ligands for transition metal catalysis [5–8] and as resolving agents [9]. Despite their high demand, the ability to produce structurally diverse chiral amines has not kept pace. Spurred by this shortcoming, innovative and improved chemical methods have been developed over the last fifteen years. Among them, nitrogen C–H insertion [10–12], hydroamination [13–16], hydroaminoalkylation [17–18], reductive amination [19–23], and enamine reduction [23–27] are experiencing a renaissance; while imine reduction [23,28–30], *N*-acylenamide reduction [23–24], and carbanion addition to imines [31–32] continue to be refined and relied on. Furthermore, enzymatic methods can offer competitive advantages that cannot be overlooked [33–35].

With this perspective, it is perhaps unsurprising that methods utilizing imines with chiral amine auxiliaries, i.e. *N*-chiral imines, can sometimes offer competitive solutions regarding the synthesis of challenging chiral amine structures [9,22]. Furthermore, it is common that alkaloid or amine containing pharmaceutical drug syntheses proceed through imine intermediates that lead to diastereomeric amine products [36–40]. With this broader perspective, insights into the variables affecting diastereoselective amine synthesis, via imines, will continue to be of importance and is the focus of this article.

## Results and Discussion

**Figure 1:**
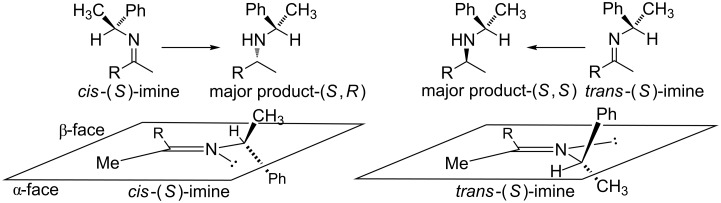
Accepted low energy conformations of the *cis*- and *trans*-imines of PEA.

**Scheme 1:**
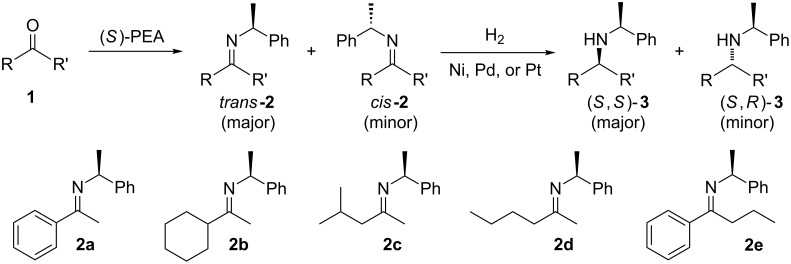
*cis*/*trans*-Phenylethylimines, their diastereomeric amine products, and the imines (**2a–e**) studied in this work.

When reducing *N*-chiral imines of (*R*)- or (*S*)-phenylethylamine (PEA), the facial preference is convincingly understood to be controlled by the phenyl group of the auxiliary (Figure 1 and Scheme 1) [36–37,39–51]. Thus the *N*-phenylethyl fragment of PEA adopts a single low-energy conformation about the nitrogen-benzylic carbon bond wherein the benzylic C–H bond is co-planar with the imine double bond and pointing toward, not away from, the imine carbonyl substituent as depicted in Figure 1 [52–53]. Accepting that facial control is enforced by the phenyl group, it is apparent from inspection of Figure 1 that reduction of the *cis*-imine will proceed preferentially from the β-face and for the *trans*-imine from the α-face. As a consequence, the diastereomeric excess (de) of the amine product correlates closely with the imine precursor’s *cis*/*trans* ratio.

**Figure 2:**
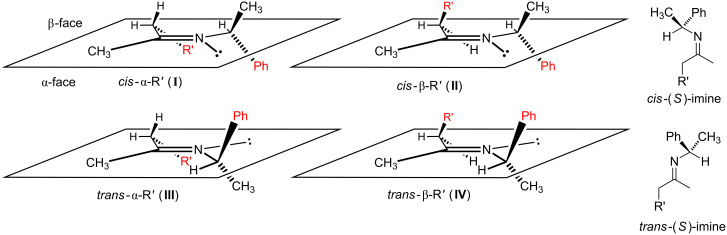
Presumed low energy conformations of α-unbranched substituted *cis*- and *trans*-(*S*)-PEA imines.

Although not previously recognized as a trend, the reduction of *N*-chiral imines possessing only mediocre *cis/trans* ratios frequently provides unexpectedly good to excellent de for the corresponding chiral amine product. An early rationale was proposed by Harada, and invokes in situ *cis*-to-*trans* imine isomerization [51]. We were curious if conformational factors may be contributing to or even dominating the stereocontrol for these apparent anomalies. In particular, we wanted to better understand if the imine carbonyl substituents of *cis*-imines, from imines with a mediocre *cis/trans* ratio, were reducing the facial selectivity (because of conformational effects) of these *N*-chiral imines, while the corresponding *trans*-*N*-chiral imines maintained ‘normal’ and high facial bias. By example, PEA imines lacking α-branching in the imine carbonyl substituent –CH_2_R (Figure 2) might suffer from eroded facial selectivity because *cis*-imine **I** would be expected to be less populated (higher in energy) than *cis*-imine **II** based solely on steric considerations (compare the proximity of the red colored R′ and Ph moieties). If true, *cis*-imine **II** (lowest energy conformation), with its α-positioned Ph moiety and β-positioned R′ moiety might suffer from reduced facial selectivity. On the other hand, the corresponding *trans*-imines **III** and **IV** (Figure 2) might be expected to be equally populated, no significant energetic difference, because the Ph moiety of the auxiliary is on the opposite side of the imine double bond, thereby removing any obvious steric interaction with R′.

If the above analysis is valid, the consequence would be enhanced selectivity for the major diastereomeric product and would explain the experimentally noted results (mediocre *cis/trans* ratio imines providing good or high ‘*trans*’ product) without invoking an in situ *cis*-to-*trans* imine isomerization. The phenomenon would be general in scope and thus allow an improved understanding of the factors controlling the stereochemical outcome of yet unknown *N*-chiral imine reductions. In this light we have investigated the stereochemical outcome for the reduction of mediocre *cis/trans* ratio imines in the presence of a heterogeneous hydrogenation catalyst and molecular hydrogen (H_2_).

For a series of (*S*)-PEA imines displaying mediocre *cis*/*trans-*imine ratios, e.g. imines **2c**–**e** (Scheme 1), we have demonstrated good or high diastereomeric amine product ratios [54]. Other researchers have recorded similar disparities, albeit for lone examples [40,47,51]. Here we formalize this fact for the first time as a recognizable trend and note it for two types of precursor ketones: methyl alkyl ketones lacking α-branching in the alkyl substituent R (Figure 1) and an aromatic alkyl ketone, i.e. phenyl *n*-propyl ketone.

### Historical perspective

Previous researchers investigating the reduction of (*R*)- or (*S*)-*N*-phenylethylimines (PEA imines) accounted for non-linear stereochemical outcomes, i.e. large differences between the starting *cis*/*trans*-imine ratios and the amine product diastereomeric ratios (dr), as occurring due to in situ *cis*-to-*trans*-imine isomerization via imine–enamine tautomerization processes. In general it was argued that tautomerization was promoted by high reaction temperatures, or the employed heterogeneous hydrogenation catalyst, or the use of a protic solvent [40,47,51]. Two of these studies are closely related to this one because they are hydrogen-based reductions employing THF/Pd-C/H_2_ and examine PEA imine reduction [47,51]. Except for Harada’s work [51], which will be discussed shortly, researchers have not offered experimental evidence to corroborate their in situ *cis*-to-*trans* imine isomerization hypotheses, they were consequently speculative [40,47]. Instead, these researchers refer back to an earlier body of literature that focused on *cis*/*trans*-imine interconversion in protic solvents or at high temperature [55–57]. A directly relevant study by Boyd et al. [55] convincingly established that protic solvents allow PEA imine isomerization, while aprotic solvents do not (see Supporting Information of the Boyd study). Of further note, when Boyd et al. did observe *cis/trans*-PEA imine ratio changes in protic solvents, they only observed increases in the *cis*-imines at the expense of the *trans*-imines. *This would be supportive of a nonlinear decrease in dr, not an increase in dr as observed here and in all other related research.* It is therefore important to note that all solvents used in our study were aprotic.

The 1972 report by Harada and co-workers [51] explored the use of (±)-PEA and (±)-phenylpropylamine (PPA) for *N*-chiral imine formation and noted their reduction with Pd-C/H_2_ in THF. For the PPA imine of phenyl ethyl ketone they stated that different ratios of the *cis/trans*-imine could be isolated. Unfortunately, they did not describe how to obtain the different *cis/trans-*imine ratios, other than to state that they change upon sitting after distillation. (Note: No temperature or vacuum settings for Harada’s distillation were noted nor were the starting or ending *cis/trans-*imine ratios noted for the reader. They did state that the *cis/trans* ratio, after distillation, returns to its original ratio after “standing for a few days”, see page 3707 of reference [51].) Reduction of this imine, regardless of the starting *cis/trans* ratio, resulted in the same amine product dr. It was then proposed that this occurred because of in situ *cis/trans* isomerization but no further evidence was offered, here we follow up on this point. Harada also noted that protic solvents can promote *trans*-imine to *cis*-imine isomerization, and he consequently observed lower drs in protic solvents, e.g. MeOH, than those found in aprotic solvents, e.g*.* benzene; this is consistent with Boyd’s findings. Subsequent to Harada’s report (1972), no researchers have reported on the ability to change the *cis/trans* ratio of (*R*)- or (*S*)-*N*-phenylethylimines, nor were we able to change the *cis/trans-*imine ratio, as judged by CDCl_3_^1^H NMR, of several of our (*S*)-*N*-phenylethylimines after heating them to as high as 65 °C for at least 12 h. (Note: It needs to be restated that Harada came to his conclusions after examining an *N*-phenylpropylimine and did not state the temperature or conditions required for the imine isomerization. Furthermore, he made no comments in his manuscript regarding the ability to change the *cis/trans-*imine ratio of the *N*-phenylethylimine he synthesized. Future researchers focused only on the use of *N*-phenylethylimines as discussed here. Additionally, we are not aware of reports after Harada’s (1972) in which researchers distilled their PEA imines and then (i) recorded their *cis/trans-*imine ratio or (ii) studied their reduction or (iii) both (i) and (ii).) All PEA-imine reductions in the present study were performed in aprotic solvent at room temperature, except for Table 1, entry 5, where the reduction was carried out at 35 °C (Table 1 and Table 2).

### Current study – background

**Scheme 2:**
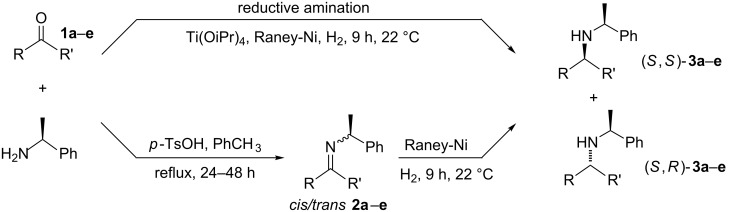
Chiral amine synthesis using (*S*)-PEA: imine reduction vs. reductive amination.

We restricted this study to the hydrogen-based reduction of imines because hydrogen is by far the most atom-economic reducing agent available. Of further advantage, we were familiar with the phenomenon (mediocre *cis*/*trans N*-chiral imine ratios yielding good to excellent amine product diastereomeric ratios) from our previous work with (*R*)- or (*S*)-PEA-based reductive amination with hydrogen [54,58–60].

(*R*)- and (*S*)-PEA are attractive chiral ammonia equivalents to study because the required imines are known [36,39–51], and the auxiliary is routinely used in either the *R* or *S* enantiomeric form on an industrial scale [9,36–37,39,61]. As a result, any conclusions from this study would have immediate usefulness. Furthermore, from the PEA-imine reduction literature [36–37,39–51], it is clear that the auxiliary is capable of inducing very high facial selectivity, and that high *trans*-to-*cis-*PEA imine ratios lead to high amine product diastereomeric ratios [41–51]. This noted, a few single outliers (mediocre *cis*/*trans*-imine ratios but unexpectedly high amine product diastereomeric ratios) have been reported in the literature [40,47,51], and this investigation focuses in particular on these type of observations. To elaborate on this idea, we examined imine **2c** (19:81, *cis*/*trans*) and imine **2d** (31:69, *cis*/*trans*). As expected, much higher amine product diastereomeric ratios were observed: **3c** (5:95, (*S*,*R*)/(*S*,*S*)) and **3d** (17:83, (*S*,*R*)/(*S*,*S*)), see Table 1 and Scheme 2. (Note: For similar discussions regarding the discrepancy of *cis/trans* chiral sulfinimine ratios and their corresponding amine product diastereomeric excesses, albeit using hydride reagents for the reduction, see the use of the (*S*)- and (*R*)-(+)-*tert*-butanesulfinamide (*t*-BuS(O)NH_2_) auxiliary sometimes referred to as: 2-methyl-2-propanesulfinamide [62]).

**Table 1:** (*S*)-PEA imine yield and geometry, imine reduction data and reductive amination data with Raney-Ni.

Entry	Ketone	Ketimine (**2**)			Diastereomeric ratio (**3)**^b^	
		
		Yield^a^ (%)	*cis/trans* ratio	Reduction solvent	Imine reduction^c^	Reductive amination^d^

1	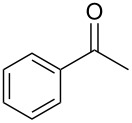 **1a**	75	6:94	EtOAc	2.5:97.5	2.5:97.5
2^e^	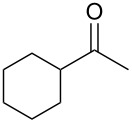 **1b**	97	6:94	MTBE	1.5:98.5	1:99
3	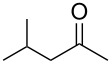 **1c**	87	19:81	hexane	5:95	3.5:96.5
4	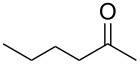 **1d**	86	31:69	DCM	17:83	17:83
5^f^	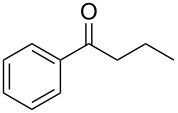 **1e**	60	32:68^g^	EtOAc	9:91	8:92^h^

^a^Crude yield, pure by ^1^H NMR. ^b^The ratios represent the (*S*,*R*)/(*S*,*S*) product ratios. ^c^The imine reductions were performed under identical reaction conditions to those of the ketone reductive amination (120 psi (8.3 bar) H_2_, Raney-Ni (100 wt %), 22 °C, 0.50 M, 9 h), except no Ti(OiPr)_4_ was added. ^d^See ref. [59–60] for reaction conditions. ^e^Reaction performed for 11 h. ^f^Reduction was performed at 35 °C for 15 h. ^g^The ratio for this imine varies from 37:63 to 32:68, see section entitled ‘An anomaly providing clarity’ for further details. ^h^See text, this is the lowest ratio observed.

We synthesized the (*S*)-PEA imines **2a-d** using a Dean–Stark trap under the conditions of refluxing toluene in the presence of catalytic quantities of *p*-TsOH (2 or 4 mol %) over 24–48 h (Scheme 2), and measured their *cis/trans-*imine ratios by ^1^H NMR (CDCl_3_). (The ^1^H NMRs are supplied in Supporting Information File 1, our chemical shift reference point was always the benzylic proton of the *cis*- and *trans*-imines. These two (one *cis*, one *trans*) down field quartets for the N-phenylethylimines are: *trans*-**2a**: 4.85 ppm, *cis*-**2a**: 4.43 ppm, *trans*-**2b**: 4.57, *cis*-**2b**: 4.75, *trans*-**2c**: 4.62, *cis*-**2c**: 4.58, *trans*-**2d**: 4.55, *cis*-**2d**: 4.59, *trans*-**2e**: 4.89, *cis*-**2e**: 4.39 ppm.) The *cis/trans* imine ratios for **2a**–**d** (Table 1) were determined after work-up (aq NaHCO_3_), drying (Na_2_SO_4_), filtration, rotary evaporation, and removal of any starting ketone or PEA by high-vacuum treatment at the elevated temperature of 50–65 °C. Imine **2e** required an alternative procedure which is discussed shortly. The procedures afforded the crude imines **2a**–**e** in good to excellent yield (75–97%) and with high purity (>97 GC area %) (Note: The *cis*-imine and *trans*-imine cannot be distinguished by GC, appearing instead as a single sharp peak. We are additionally unaware of any reports detailing the GC separation of PEA-*cis/trans*-imines.) Furthermore, the unhindered nonaromatic ketones could also be converted to the same imines by stirring the ketone and (*S*)-PEA in anhydrous methanol (no acid additive) for 24 h at 25 °C. For example, imine **2c**, after concentration and high vacuum treatment at 40 °C (removes PEA), was isolated in 44% yield with high purity (>97 GC area %). Regardless of whether this ‘low temperature’ protocol was used or the ‘high temperature’ Dean–Stark procedure (refluxing toluene), the *cis/trans*-imine ratio was found to be consistent and equivalent for both methods.

The following points refer to amine product **3e**. In reference [60], Table 4 (entry 3) and Figure 4 show the reductive amination product **2j** (which is equal to amine **3e** in this manuscript) as having a 94% de. A more in depth examination has shown that when reducing the isolated imine precursor to amine **3e**, the observed de has consistently been 82%. When subsequent reductive amination procedures were applied we found de ranges from 84–92% de. These numbers are significantly lower than found in our original report [60], see Table 3 for further information.

With the synthesis of the imines clarified, we sought to gain insight regarding the *cis/trans* ratio during the formation of the imines via NMR. Reliable integration of the signals due to the minor *cis*-imine, from the background noise, was only possible after heating isobutyl methyl ketone (**1c**) and (*S*)-PEA, in equamolar ratios, in toluene-*d*_8_ (1.8 M) in the presence of *p*-TsOH (25 mol %) at 40 °C over 24 h. (Note: Attempts with CDCl_3_ failed to show significant imine formation (<5%), the solvent used for the reduction of imine **2c** is hexane. Further note that reactions employing Ti(OiPr)_4_ (instead of a Bronsted acid) were not used for in situ ^1^H NMR analysis because of stark peak broadening.) Subsequent recording of the room temperature sample provided a *cis/trans* ratio of 18:82 (toluene-*d*_8_, 22 °C), which is very similar, 19:81 (CDCl_3_, 22 °C), to that found after Dean–Stark trap synthesis in toluene followed by workup and high vacuum drying. We also examined the effect of heating imine **2c** (synthesized and isolated in pure form after Dean–Stark trap synthesis) in an NMR tube for 75 min at 60 °C, and then recording its ^1^H NMR at 60 °C. Even at this elevated temperature the *cis/trans*-imine ratio remained static in the examined aprotic NMR solvents (CDCl_3_ and toluene-*d*_8_). Using a similar approach the ^1^H NMR for imine **2d**, from 2-hexanone (**1d**), showed the same *cis/trans-*imine ratio after being maintained for 75 min at each of three increasingly higher temperatures: −15 °C, 22 °C, and finally 38 °C, in CD_2_Cl_2_. At the end of each 75 minutes, the ^1^H NMR data was collected at the indicated holding temperature. This was an important result because the optimal solvent for the reduction of imine **2d** is CH_2_Cl_2_. Of further note, the *cis/trans-*imine ratio of **2d** was the same in CD_2_Cl_2_ and CDCl_3_.

*Cis*-to-*trans* isomerization via surface based phenomena on the heterogeneous hydrogenation catalysts were also examined. Stirring imines **2a**–**e** under the optimized reaction conditions, albeit lacking pressurized hydrogen, resulted in no change in the *cis*-to-*trans* ratio of the imines. This was true regardless of whether the hydrogenation catalyst (Pd, Pt, Raney-Ni) was pre-activated with H_2_ or not (for further details, see Supporting Information File 1). (Note: The reduction of a small number of cyclic imines with Pd/C has been reported, and showed a significant positive discrepancy between the *cis/trans* ratio of the imine and the corresponding product diastereomeric ratio – see reference [40]. In that study, the non-linearity is explained by referring back to Harada’s study [51] as discussed in the Historical Perspective section, to invoke a dynamic kinetic resolution. Furthermore, in a further effort to investigate the possibility of metal catalyst induced isomerization, we reduced imines **2a–e** using significantly higher loadings of Pd/C or Pt/C at 22 °C than noted in Table 2, but found no inconsistency pointing to isomerization.)

These overall findings are consistent with our earlier observations regarding phenylethylimine *cis*/*trans* ratios, i.e. regardless of how they are synthesized (toluene reflux and isolation, room-temperature stirring in anhydrous methanol and isolation, or formed in situ (^1^H NMR study) or whether they are stirred with a heterogeneous hydrogenation catalyst or not), the *cis/trans-*imine ratios were found to be static in the aprotic reaction solvents we measured them in.

### Reduction of imines **2**

The reported *cis*/*trans* ratio (5:95) for imine **2a** [55,63–64], from acetophenone (**1a**), is very similar to our own measurement (6:94). (Regarding the Hogeveen work [47], please note the following: The ^1^H NMR assignment of the *cis*-imine **2a** (this article’s numbering system) was mistaken for a resonance pattern originating from unremoved starting material (PEA). The chemical shift of PEA’s quartet is 4.01 ppm. The commonly used method for *cis/trans-*imine ratio assessment is to compare the two down field quartets originating from the PEA auxiliary. See the ^1^H NMR data for imine **4a** (Table 1) of ref [47]. Hogeveen reduced the (*S*)-*N*-phenylethylimine of acetophenone with Pd-C/H_2_ and recorded a 70% de. This is much lower than what we and others have observed (95% de). Compare Hogeveen’s Scheme 1 and Table 1 & 2, with Table 3 of this article to clarify their error. It is noteworthy that Hogeveen’s article also focused on conformational effects, albeit different from the ones discussed in this work, to explain the non-linear stereochemical outcome of *N*-phenylethylimine reduction aryl-alkyl ketone precursors.) Nevertheless, no prior evidence was provided to establish the *cis* or *trans* assignment of the major imine of **2a**. Using NOESY experiments we have unambiguously identified the major imine isomer as being *trans* for all of the (*S*)-*N*-phenylethylimines (**2a–e**) examined here (Table 1 and Supporting Information File 1).

With the *cis/trans* ratio of imines **2a**–**e** defined (^1^H NMR) and the major isomer for all imines unequivocally established as *trans*, we then proceeded to investigate the dr resulting from their reduction. We previously reported the asymmetric reductive amination of all precursor ketones **1a–e** in the presence of Ti(OiPr)_4_, Raney-Ni and hydrogen (8.3 bar, 120 psi) at room temperature in a variety of aprotic solvents [54,58–59]. For the current study we isolated the corresponding imines, as noted above, and reduced them under similar reaction conditions (Scheme 2). The amine product diastereoselectivity was the same as when the corresponding ketone was reductively aminated with (*S*)-PEA in the presence of Ti(OiPr)_4_ (Table 1, compare last two columns). To the best of our knowledge Table 1 (entries 3–4) shows the first examples of acyclic aliphatic *N*-phenylethylimine reduction with molecular hydrogen, and the experimental data represents some of the largest differences in the *cis*/*trans*-imine ratios to the amine product diastereomeric ratios ever noted. (Note: Compare with the earlier reports for aryl alkyl ketones, see [41,48].)

The dr of amine products **3a–e** and the starting *cis/trans-*imine ratios correlate reasonably well when using Pd or Pt heterogeneous hydrogen catalysts, implying that isomerization, if occurring, is minimal. On the other hand, reduction of the same (*S*)-PEA imines under Raney-Ni/H_2_ catalysis consistently provided significantly higher drs (Table 2). Based on these Pt, Pd, and Ni results two important conclusions were arrived at: i) on average, when using an active-surface catalyst such as Pd/C or Pt/C, the *trans*-imine provides the major diastereomer, (*S*,*S*)-**3**, while the *cis*-imine the minor diastereomer (*S*,*R*)-**3** (Scheme 1). Hence, the dr reflects, to a broad degree, the native *cis/trans-*PEA imine ratio (Table 2); and ii) for imines lacking α-branching in the carbonyl substituent, mediocre *cis/trans* ratios are observed, yet Raney-Ni is capable of converting them into amine products with greatly improved diastereomeric ratios (Table 2, entries 3–5). The disparity between the highly-reactive Pd and Pt catalysts (0.5–1.0 mol % loading) and the less-reactive Raney-Ni (100 wt %) are doubtless a consequence of the well-known relationship between reactivity and selectivity. In support of this contention, we found that at lower Pd or Pt catalyst loadings, or even with poisoned Pd catalysts (specifically Lindlar’s catalyst), an increasing trend of higher diastereoselectivity was noted, albeit without complete reaction (see Supporting Information File 1, Tables S1–7). Examination of Rosenmund’s catalyst resulted in recovery of the imine [65–66].

**Table 2:** (*S*)-PEA imine reduction with Raney-Ni, Pd/C and Pt/C.

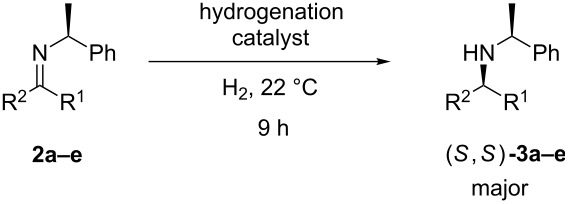					

			Diastereomeric ratio of amine **3a–e** (corresponding de of **3a–e** )		

Entry	Imine structure	*cis/trans* imine ratio	Raney-Ni^a^	Pd/C^a^	Pt/C^a^

1	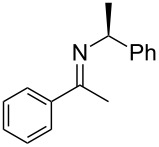 **2a**	6:94	2.5:97.5 (95)	11.5:88.5 (77)	11.5:88.5 (77)
2	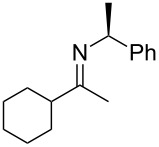 **2b**	6:94	1.5:98.5 (97)^b^	9.5:90.5 (81)^b^	11.5:88.5 (77)^b^
3	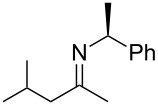 **2c**	19:81	5:95 (90)	13:87 (74)	20:80 (60)
4	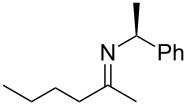 **2d**	31:69	17:83 (66)	31:69 (38)	34.5:65.5 (31)
5	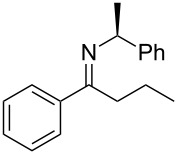 **2e**	32:68	9:91 (82)^c^	15.5:84.5 (69)^c^	33:67 (34)^c^

^a^100 wt % Raney-Ni or 0.5 mol % of Pd or Pt were used. ^b^Reaction performed for 11 h. ^c^Reaction performed for 15 h at 35 °C.

### An anomaly providing clarity

Our discussion has generally avoided imine substrate **2e**, the imine of phenyl *n*-propyl ketone (butyrophenone). Late in our study, we recognized that this imine provided non-robust diastereomeric data. The starting point for that observation was its synthesis. This more hindered ketone required demanding reaction conditions (see Supporting Information File 1), with perhaps the important change being the final neat purification under high vacuum for 24 h at the elevated temperature of 80 °C (to remove the remaining, and high boiling, butyrophenone) before the *trans/cis-*imine ratio was measured at room temperature (CDCl_3_, ^1^H NMR). (Work-up and purification were as follows: EtOAc and aqueous NaOH (1.0 M) were added and stirred for 1 h. Then separatory funnel separation and further extraction was performed. The EtOAc layers were combined, concentrated, and treated under high vacuum at 80 °C for 24 h to remove the starting ketone, butyrophenone, and the (S)-PEA. It is important to note that the maximum temperature the other imines (**2a–d**) were exposed to was 65 °C during neat high vacuum drying.) Four separate batches of this imine were synthesized and each had its *trans/cis* ratio measured within 2 h after being removed from the 80 °C heat source. This resulted in a tight range for the *trans/cis* data: 63.5:36.5 (±1 unit). Imine **2e** prepared in this way was then stored under nitrogen and monitored on a daily basis using ^1^H NMR. After four days at room temperature, a *trans/cis* resting point of 67.5:32.5 (±1 unit) was achieved, and no discernible change was noted when the monitoring was extended to 7 d. This observable change in the *trans/cis-*imine ratio is in stark contrast to those of imines **2a–d**, for which no *trans/cis* ratio changes were ever observed. One sample of imine **2e**, that was equilibrated over a 5 d period at room temperature, was then heated neat to 80 °C (under high vacuum), its *trans/cis* ratio changed from 67.9:32.1 (before heating) to 63.5:36.5 within 5 h of heating at 80 °C. This demonstrated a clear, and reversible, thermal dependence of the *trans/cis-*imine ratio of **2e**. Hydrogenation of the butyrophenone-imine **2e** with different *trans/cis* starting ratios provided the reduction product with the same diastereoselectivity (Table 3). It can be concluded that the imine equilibrates during the reaction and that Harada’s hypothesis concerning in situ *cis*-to-*trans* transition metal-induced isomerization is operative for imine **2e** and by extension for all of the examples noted here with Raney-Ni.

**Table 3:** Imine **2e** (Scheme 1) reduction product profiles using different starting *trans/cis* ratios.^a^

Entry^a^	Imine *trans/cis* ratio	dr, Raney Ni (100 wt %)^b,c^	dr, Pt/C (0.5 mol %)^b^

1	63.7:36.3	91.1:8.9	68.4:31.6
2	62.9:37.1	90.5:9.5	65.8:34.2
3	63.5:36.5	90.8:9.2	66.4:33.6
4	67.3:32.7	91.7:8.3	67.8:32.2
5	67.2:32.8	91.0:9.0	68.3:31.7
6	67.6:32.4	90.8:9.2	67.2:32.8

^a^Reactions performed using 8.3 bar H_2_, 35 °C, in 0.50 M EtOAc, for 15 h. ^b^The amine product ratios are expressed as *trans*/*cis* as found in CDCl_3_ by ^1^H NMR integration. ^c^100 wt % is in reference to the starting imine.

The isomerization of imine **2e** significantly undermines the hypothesis that conformation may be playing a significant and previously unappreciated role in the stereochemical outcome of *N*-chiral imine reductions. Further corroborative support for the Harada isomerization hypothesis came from the examination of the low-energy conformations of our imines via DFT analysis. To achieve this, we first imposed the accepted conformation of the auxiliary, Figure 1 (vide supra) [52–53], and then turned our attention to the likely conformations of the carbonyl substituents. Due to allylic 1,2-strain [52], the –R' group of the –CH_2_R' carbonyl substituent (Figure 2) is expected to avoid eclipsing interactions with the other imine carbonyl substituent (methyl group), thus the –R' group will either reside above or below the plane of the imine double bond and will point in the direction of the auxiliary. (Note: For a similar analysis, albeit for α-branched substrates where R of Figure 1 equals –CHR'_2_, see Supporting Information File 1.) With these points in mind, examination of the low energy imine conformations was either not supportive or only weakly supportive of a conformational effect originating from the imine carbonyl substituent.

## Conclusion

*Cis*→*trans* imine isomerization is the accepted rationalization proposed for the observed non-linear relationship between imine *cis*/*trans* ratios and their reduction product (chiral amine) diastereomer ratios. Density functional theory modeling of the experimentally explored imines discussed in this paper did not support the alternative hypothesis that we set out to consider, namely that the carbonyl substituent R of a *cis*-phenylethyl-imine (Figure 1) is capable of reducing the facial selectivity imposed by the phenyl group of the PEA auxiliary, while the same carbonyl substituent would not impose a reduction in facial selectivity for the *trans*-*N*-phenylethylimine.

## Supporting Information

File 1 Experimental and analytical data.
