# Supplementary material for: An investigation of the observed, but counter-intuitive, stereoselectivity noted during chiral amine synthesis via N-chiral-ketimines
Source: Beilstein J Org Chem. 2013 Oct 15;9:2103–12. doi: 10.3762/bjoc.9.247 (PMC3817511; doi:10.3762/bjoc.9.247)
Supplement: File 1 — Experimental and analytical data. [file Beilstein_J_Org_Chem-09-2103-s001.pdf]

Supporting Information

for

An investigation of the observed, but counter-intuitive, stereoselectivity noted during chiral amine synthesis via *N*-chiral-ketimines

Thomas C. Nugent*[1], Richard Vaughan Williams*[2], Andrei Dragan[1], Alejandro Alvarado Méndez[1], and Andrei V. Iosub[1]

[1]Department of Chemistry, School of Engineering and Science, Jacobs University Bremen, Campus Ring 1, 28759 Bremen, Germany and [2]Department of Chemistry, University of Idaho, PO Box 442343, Moscow, ID 83844-2343, USA

E-mail: Thomas C. Nugent* - t.nugent@jacobs-university.de and Richard V. Williams* - williams@uidaho.edu

Experimental and analytical data.

General experimental details

NMR spectra were recorded on JEOL ECX 400 spectrometer, operating at 400 MHz (^{1}H) and 100 MHz (^{13}C) respectively. Chemical shifts were reported in parts per million (ppm) downfield from TMS (=0) or in the scale relative to $CHCl_3$, 7.24 ppm for ^{1}H NMR and 77.00 for ^{13}C NMR. Multiplicities are abbreviated as: s, singlet; d, doublet; q, quartet; m, multiplet; br, broad. Coupling constants are measured in Hertz. For imine and amine products, reaction progress and dr measurements were obtained using a Shimadzu GC-2010 instrument with a Rtx-5 amine column (Restec, 30m x 0.25mm); T_{inj} = 300 °C and T_{det} = 300 °C were always constant; Program A: 50 °C (hold 1 min), 280 °C (20 °C/min,

then hold 5 min), Program B: 50 °C (hold 1 min), 280 °C (14 °C/min, then hold 5 min) and Program C: 50 °C (hold 1 min), 100 °C (5 °C/min), then up to 280 °C (40 °C/min, then hold 2 min). All reagents were obtained from Sigma-Aldrich and used without further purification. 97% grade $Ti(OiPr)_4$ (catalog number, 205273) was used for all screening reactions (2–5 mmol). (*S*)-PEA (catalog number, 115568) was of 98% chemical purity and 98% ee. Platinum 5% on activated carbon (catalog number, 205931), Palladium 5% (dry basis) on activated carbon 50% water (catalog number, 276707), Palladium 5% on $CaCO_3$ with Lead (Lindlar's catalyst) (catalog number, 62145), Palladium on barium sulfate 5% Pd (catalog number, 76022) were all used as heterogeneous hydrogenation catalysts. The Raney-Nickel (in water) was purchased from Fluka (catalog number, 83440). All of the hydrogenation reactions were performed with reagents kept under nitrogen atmosphere and anhydrous solvents.

Conformation based hypothesis – extended discussion

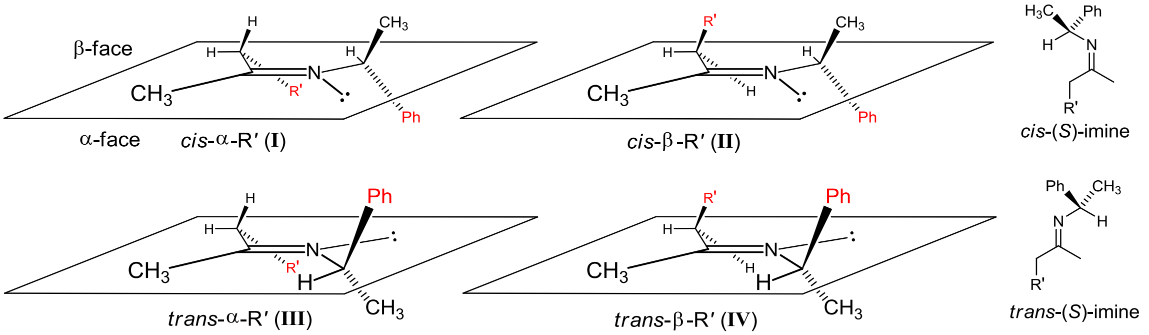

Figure 2 (as copied from the manuscript).

When considering the $–CH_2R'$ carbonyl substituents (Figure 2 above), no α-branching is present, e.g. *n*-butyl (imine **2d**) or ibutyl (imine **2c**), it is apparent that in the *cis* isomer steric interactions between the PEA phenyl group and the R' group will favor the *cis*-β-R' (II) conformation over the *cis*-α-R' (I) conformation. This stereochemical relay effect should have the consequence of eroding the expected facial selectivity for the *cis*-imine (if *cis*-α-R' (I) and *cis*-β-R' (II) are reduced at similar reaction rates. In the corresponding *trans*-imine series (Figure 2), the effect is expected to be minimal or non-existent due to a lack of proximity between the Ph and R' groups, i.e. conformations *trans*-β-R' (IV) and *trans*-α-R' (III) would be expected to be similar in energy. We therefore proposed that *cis*-*N*-phenylethylimines might experience facial erosion, or even inversion of facial selectivity, due to a strong steric effect imposed by the phenyl group of PEA on the proximal carbonyl substituent. In the corresponding *trans* series the effect is minimal.

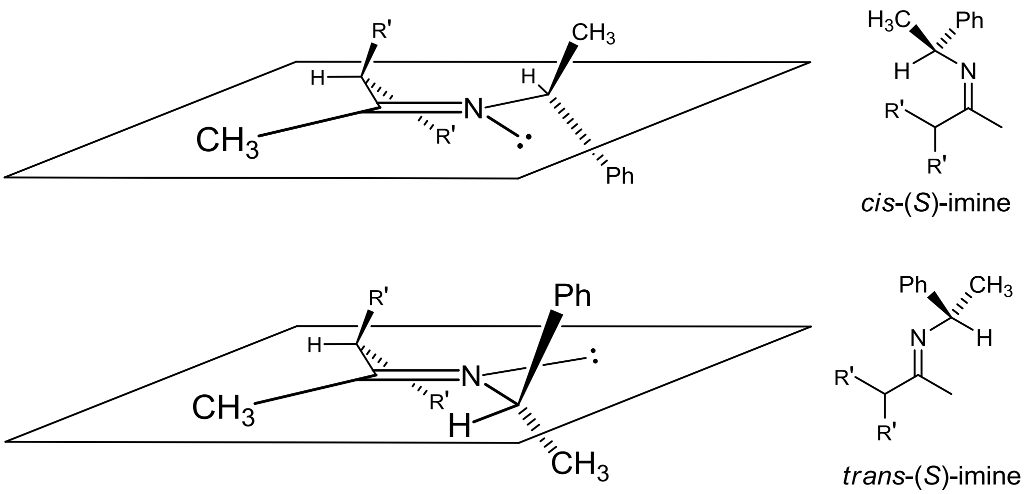

Figure S1: Presumed low energy conformations of α-branch substituted *cis*- and *trans*-(*S*)-PEA imines.

On changing to α-branched carbonyl substituents, –CHR'$_2$ (Figure 1S, above), e.g. *c*-hexyl (imine **2b**) and phenyl (imine **2a**), the conformational implications change dramatically. Again, allylic 1,2-strain is expected to force the α-hydrogen of the -C<u>H</u>R'$_2$ substituent to be eclipsed with the methyl group (Figure A). This conformation has the effect of locating one R' substituent on one face of the imine double bond and the other R' group on the opposite face of the imine double bond. When the R' groups are equivalent, both faces of the imine double bond would be similarly hindered. An immediate consequence would be little or no change in the 'expected' facial selectivity for both the *cis*-imine and *trans*-imine. Thus carbonyl substituents with α-branching should largely lead to a linear relationships between *cis/trans* imine ratios and the product amine diastereomeric ratios, and our experimental data strongly supported this conclusion.

Extra tabular information follows

Table S1: Effect of stirring the hydrogenation catalyst (hydrogen-activated and non-activated) and (*S*)-phenylethylimines in the absence of hydrogen.

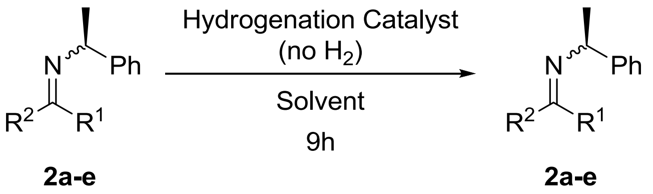

Entry	Ketimine	Solvent	Catalyst[c,d]	*cis/trans* ratio		
				Before exposure	After exposure	
					Non-activated	Hydrogen-Activated[a]

1			Pd/C		6:94	5:95
2	**2a**	EtOAc	Pt/C	6:94	5:95	5:95
3			Raney-Ni		6:94	5:95
4			Pd/C		7:93	7:93
5	**2b**[b]	MTBE	Pt/C	6:94	7:93	7:93
6			Raney-Ni		7:93	7:93
7			Pd/C		20:80	19:81
8	**2c**	Hexane	Pt/C	19:81	18:82	17:83
9			Raney-Ni		19:81	19:81
10			Pd/C		32:68	34:66
11	**2d**	DCM	Pt/C	31:69	30:70	31:69
12			Raney-Ni		28:72	25:75
13			Pd/C		32:68	31:69
14	**2e**[b]	EtOAc	Pt/C	32:68	30:70	32:68
15			Raney-Ni		31:69	32:68

[a]Catalyst was activated by treatment with H_2 at 120 psi for 24 h in the indicated solvent before stirring with the imine. [b]Substrates **2b** and **2e** were stirred for 11 h and 15 h respectively. Ketimine **2e** was stirred at 35 °C, in accordance to the reductive amination temperature protocol. [c]100 wt % Raney-Ni was used. [d]Catalyst loading of 0.5 mol %.

Palladium tables

Table S2: Reduction conditions and results for imine **2d**.

**Catalyst**	**Loading (mol %)**	**Conversion 9 h (%)** [a]	**de 9 h (%)** [b]	**Conversion 24 h (%)** [a]	**de 24 h (%)** [b]
Pd/C [c]	0.50	36	38	46	40
Pd/C [d,e]	0.50	79	41.5	82	41.5
Pd/$CaCO_3$ poisoned with lead (Lindlar)	0.50	27.3	51.5	28	52
Pd / Alumina	0.50	43	42	51	41

Table S3: Reduction conditions and results for imine **2c.**

**Catalyst**	**Loading (mol %)**	**Conversion 9 h (%)** [a]	***de* 9 h (%)** [b]	**Conversion 24 h (%)** [a]	***de* 24 h (%)** [b]
Pd/C [c,e]	0.50	25	74	50	72
Pd/C [d,e]	0.50	24.3	76.3	32	76.3
Pd/$CaCO_3$ poisoned with lead (Lindlar)	0.50	18.5	82	23.4	82
Pd/$CaCO_3$ poisoned with lead (Lindlar)	0.10	0.3	78.6	0.32	78.6

Table S4: Reduction conditions and results for imine **2e.**

Catalyst	Loading (mol %)	Conversion 15 h (%) [a]	*de* 15 h (%) [b]
Pd/C [c]	0.50	74.5	69.7
Pd/C [c]	0.10	71	85
Pd/C [d,e]	0.50	73	79
Pd/$CaCO_3$ poisoned with lead (Lindlar)	0.50	47	84
Pd/$CaCO_3$ poisoned with lead (Lindlar)	0.10	48	91

[a] determined by GC, based on ketone, imine and amine product peaks
[b] determined by GC
[c] used as purchased from Sigma-Aldrich, 50% wet
[d] dried under high vacuum overnight at 50°C
[e] conversion was below 10 area % product at 0.1 mol% loading

Platinum tables

Table S5: Reduction conditions and results for imine **2d.**

Catalyst	Loading (mol %)	Conversion 9 h (%) [a]	de 9 h (%) [b]	Conversion 24 h (%) [a]	*de* 24 h (%) [b]
Pt/C [c]	0.50	96.5	31	-	-
Pt/C [c]	0.10	85	32.5	86	32.5
Pt/C [c]	0.05	58	34	60	34

Table S6: Reduction conditions and results for imine **2c.**

Catalyst	Loading (mol %)	Conversion 9h (%) [a]	de 9h (%) [b]	Conversion 24 h (%) [a]	*de* 24 h (%) [b]
Pt/C [c]	0.50	full	60	-	-
Pt/C [c]	0.10	full	60	-	-
Pt/C [c]	0.05	95%	59	full	59
Pt/C [d]	0.50	full	64	-	-

Table S7: Reduction conditions and results for imine **2e.**

Catalyst	Loading (mol %)	Conversion 15 h (%) [a]	*de* 15 h (%) [b]
Pt/C [c]	0.50	full	34.3
Pt/C [c]	0.10	full	36.5

[a] determined by GC, based on ketone, imine and amine product peaks
[b] determined by GC
[c] used as purchased from Sigma-Aldrich, dry
[d] 50% wet and then dried under high vacuum overnight at 50°C

Synthesis of the Rosenmund catalyst (Pd/$BaSO_4$ poisoned with quinoline-sulfur)

1.0 g of sulfur (Sigma-Aldrich, catalog number, 215198) and 5.0 g of quinoline (Sigma-Aldrich, catalog number, 453544) were refluxed for 5 h. The resulting dark brown liquid was diluted to 70 mL with xylene. To that solution was added an equimolar quantity of Pd/$BaSO_4$ as compared to the quinoline. This procedure was adapted from references [1S,2S].

General procedure for asymmetric reductive amination

In an anhydrous solvent (0.50–0.60 M) a prochiral ketone **1** (2.5 or 5.0 mmol), titanium tetraisopropoxide (1.25 equiv), and (*S*)-PEA (1.10 equiv) were combined and stirred at room temperature for 30 min. Raney-Ni (100 wt %; the catalyst was first triturated with EtOH (x 3) and then with the anhydrous reaction solvent (3 x 0.5 mL) before addition to the reaction) was then added and the vessel pressurized at 120 psi (8.3 bar) of hydrogen. After 9 h, the reaction was stopped and the mixture was stirred with aqueous NaOH (1.0 M, 10 mL) for 1 h. The heterogeneous mixture was then filtered through a bed of celite, and the celite washed with CH_2Cl_2 or EtOAc. The filtrate was concentrated (rotary evaporator) to remove the low boiling organics and the remaining aqueous solution was then extracted with CH_2Cl_2 (3 x 15 mL). The combined organic extracts were dried (Na_2SO_4), filtered, concentrated (rotary evaporator), and GC analysis was performed to determine chemical purity and de. For isolated yield data see reference [S3].

Synthesis of (*S*)-phenylethylimine (2c) in methanol at room temperature

Ketone **1c** (6.00 mmol) and (*S*)-PEA (1.10 equiv) are added to a round bottom flask containing anhydrous methanol (0.80 M). The reaction mixture was stirred for 24 h at room temperature. Concentration of the mixture (rotary evaporation) and high vacuum drying yields the respective ketimine (534 mg, 44% crude yield, > 98% GC purity).

Experimental procedure for (*S*)-phenylethylimine reduction

The (*S*)-PEA(**2a**–**e**) (1.25 mmol) was added to an organic solvent as indicated in the text of this article (0.60 M). Raney-Ni (100 wt %, triturated prior addition three times with EtOH and three further times with the reaction solvent) or Pt/C (0.50 mol %) or Pd/C (0.50 mol %) or Pd/$CaCO_3$ with Lead (Lindlar's catalyst, 0.50 mol %) was then added, and the mixture was hydrogenated at room temperature under 8.3 bar of hydrogen for 9 h (unless specified otherwise in the text). As a precautionary measure work up involved stirring with aqueous NaOH solution (1.0 M, 20.0 mL) for 1 h to ensure that any traces of the ketimine were hydrolyzed. The heterogeneous mixture was then filtered through a bed of celite, and the celite washed with CH_2Cl_2 or EtOAc. The filtrate was concentrated (rotary evaporator) to remove the low boiling organics and the remaining aqueous solution was extracted with CH_2Cl_2 (15 mL x 3). The combined organic extracts were dried (Na_2SO_4), filtered, concentrated (rotary evaporator), and GC analysis was performed to determine chemical purity and de.

Deuteration of (*S*)-shenylethylimine

Under a nitrogen atmosphere the (*S*)-phenylethylimine of **2a** or **2c** (1.0 mmol) is added to a vessel by means of a syringe, followed by CD_3OD (0.80 M). The mixture is stirred for 12 h and then concentrated (rotary evaporation and then under high vacuum) at room temperature. Multiple deuteration was confirmed by ^1H NMR analysis.

Effect of heterogeneous hydrogenation catalyst on (*S*)-phenylethylimines 2a–e

Catalyst activation: The hydrogenation catalyst (for Raney-Ni, trituration with EtOH (x 3) and the reaction solvent (x 3) with the reaction solvent) was deposited in a hydrogenation vessel with the reaction solvent. The vessel was then pressurized with hydrogen (120 psi, 8.3 bar) and stirred for 9 h. Concentrations and temperatures were in accordance with the imine reduction protocol.

(*S*)-Phenylethylimine **2a-e** (1.0 mmol) was added under nitrogen atmosphere to a hydrogenation vessel containing the reaction solvent (0.60 M) and the hydrogenation catalyst (activated or non-activated) by means of a syringe, in the same amounts as called for by the imine reduction protocol. The mixture was left to stir for 10 h and then filtered and concentrated under high vacuum for 6 h at room temperature. *cis/trans* Ratios were determined by ^1H NMR analysis of this material.

General procedure for the preparation of (*S*)-phenylethylimines 2a–d

p-Toluensulfonic acid (2.00 or 4.00 mol %) was added to a double-neck 100 mL round bottom flask equipped with a Dean–Stark trap (the water collection arm had spherical 4 Å molecular sieves added to it) and reflux condenser. To this was added toluene (0.50 M), ketone (32.00 mmol), and (*S*)-PEA (1.10 equiv). The mixture was refluxed for 24–60 h, then cooled to room temperature and concentrated (rotary evaporator). The residue was dissolved in hexane (or CH_2Cl_2) and filtered to remove the precipitated *p*-TsOH, and the hexane briefly washed with aqueous $NaHCO_3$ (1.0 M, 40 mL). The hexane was then washed with brine solution, dried over $MgSO_4$, filtered, and concentrated (rotary evaporator). The resulting oil was then dried under high vacuum at 50 °C (unless specified otherwise) for 24 h to remove any starting materials giving the crude product. GC analysis of the crude product showed less than 2% of the starting ketone and <1% (*S*)-PEA. *cis/trans* Ratios were determined on the crude product by ^1H NMR.

Note: none of the *cis* and *trans*-imines are separable by GC.

Below specific examples of (*S*)-phenylethylimines synthesized by this method are presented.

(*S*)-1-Phenyl-*N*-(1-phenylethylidene)ethanamine (2a)

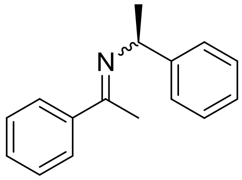

Reaction details: Acetophenone, 0.50 M; reaction time: 24 h; (5.43 g) 76% crude yield. The mixture of *cis*- and *trans*-isomers was isolated as a yellow oil after high vacuum drying. GC (program A, see General experimental details) retention time [min]: major ***(S)-trans*-2a** and minor ***(S)-cis*-2a** isomers, 12.01.
^{1}H NMR (400 MHz, $CDCl_3$): 7.85–7.08 ppm (m, 10H), 4.85 (*trans*, q, *J* = 6.7 Hz, 1H), 4.43 (*cis*, q, *J* = 6.5 Hz, 1H), 2.32 (*cis*, s, 3H), 2.27 (*trans*-, s, 3H), 1.53 (*trans*, d, *J* = 7.1 Hz, 3H), 1.40 (*cis*, d, *J* = 6.7 Hz, 3H). See references [S4-8].

(*S*)-*N*-(1-Cyclohexylethylidene)-1-phenylethanamine (2b)

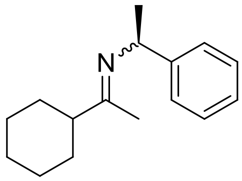

Reaction details: *c*-Hexylmethyl ketone, 0.50 M; reaction time: 24 h; (7.12 g) 97% crude yield. The mixture of *cis*- and *trans*-isomers was isolated as a dark yellow oil after high vacuum drying. GC (program B, see General experimental details) retention time [min]: major (*S*)-*trans*-**2b** and minor ***(S)-cis*-2b** isomers, 14.89.
^{1}H NMR (400 MHz, $CDCl_3$): 7.35–7.27 ppm (m, 5H), 4.75 (*cis*, q, *J* = 6.7 Hz, 1H), 4.57 (*trans*, q, *J* = 6.8 Hz, 1H), 2.22 (br. s, 1H), 1.96 (*cis*, s, 3H), 1.76 (*trans*, s, 3H), 1.75(m, 4H), 1.43 (d, *J* = 6.8 Hz, 3H), 1.41-1.30 (m, 6H).

(*S*)-*N*-(4-Methylpentan-2-ylidene)-1-phenylethanamine (2c)

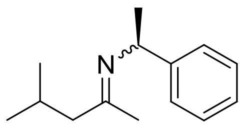

Reaction details: 4-Methyl-2-pentanone, 0.50 M; reaction time: 16 h; (5.72 g) 88% yield. The mixture of *cis*- and *trans*-isomers was isolated as a yellow oil after high vacuum drying. GC (program A, see General experimental details) retention time [min]: major (*S*)-*trans*-**2c** and minor (*S*)-*cis*-**2c** isomers, 9.70.
^{1}H NMR (400 MHz, $CDCl_3$): 7.35–7.27 ppm (m, 5H), 4.62 (*cis*, q, *J* = 6.8 Hz, 1H), 4.58 (*trans*, q, *J* = 6.7 Hz, 1H), 2.18 (d, *J* = 7.4 Hz, 2H), 2.03 (*cis*, s, 3H), 2.00 (m, 1H), 1.81 (*trans*, s, 3H), 1.47 (*trans*, d, *J* = 6.8 Hz, 3H), 1.44 (*cis*, d, *J* = 6.6 Hz, 3H), 0.90 (m, 6H).

(*S*)-*N*-(Hexan-2-ylidene)-1-phenylethanamine (2d)

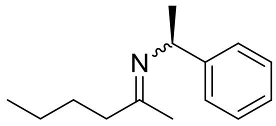

Reaction details: 2-Hexanone, 0.50 M; reaction time: 24 h; (5.55 g) 86% yield. The mixture of *cis*- and *trans*-isomers was isolated as a yellow oil after high vacuum drying. GC (program C, see General experimental details) retention time [min]: major (*S)-trans*-**2d** and minor ***(S)-cis*-2d** isomers, 15.31.
^{1}H NMR (400 MHz, $CDCl_3$): 7.35–7.27 ppm (m, 5H), 4.59 (*cis*, q, *J* = 6.8 Hz, 1H), 4.55 (*trans*, q, *J* = 6.4 Hz, 1H), 2.28 (m, 2H), 2.03 (*cis*, s, 3H), 1.82 (*trans*, s, 3H), 1.46 (m, 2H), 1.45 (*cis*, d, *J* = 6.4 Hz, 3H), 1.44 (*trans*, d, *J* = 6.5 Hz, 3H), 1.33 (m, 2H), 0.91 (t, *J* = 7.3 Hz, 3H).

(*S*)-1-Phenyl-*N*-(1-phenylbutylidene)ethanamine (2e)

The synthesis of imine **2e**, as noted in the main text is different from that of imines **2a–d**.

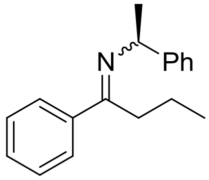

A representative example follows. To a 150 mL double neck round bottom flask containing EtOAc (0.75 M, 32 mL), the following were added under nitrogen in this order: $Ti(OiPr)_4$ (1.25 equiv, 30 mmol, 8.88 mL), butyrophenone (1.00 equiv, 24 mmol, 3.48 mL), (*S*)-PEA (1.10 equiv, 26.4 mmol, 3.37 mL). The mixture was stirred for 6 h at room temperature, and then quenched by stirring with aqueous NaOH (1.0 M, 30 mL) for 1 h. [Note: although this imine is reasonably resistant to these hydrolysis conditions, this cannot be said for the other imines studied here. If imines **2a-d** are synthesized in this manner the work-up would require the less basic conditions of Na_2CO_3 (0.5 M) and with shortened exposure times, e.g. 15 min. The basic conditions convert $Ti(OiPr)_4$ trapped intermediates and products to TiO_2, without this filtration and sedimentation is frequently observed with reduced yields] The organic phase was collected and the aqueous phase was again extracted with EtOAc (3 x 30 mL). All of the organic extracts were combined and dried (Na_2SO_4), filtered, concentrated (rotary evaporator), and the resulting oil was placed under high vacuum at 80 °C for 24 h. [Note 80 °C was required to remove the high boiling butyrophenone (bp 228–230 °C). The yields ranged from 55-70%, with 60% occurring most often, 2.26 g). These low yields imply imine hydrolysis during the stirring with aqueous NaOH. Again, the *cis*/*trans* ratio was determined using the crude product by ^{1}H NMR. GC (program B, see General Experimental Details) retention time [min]: major ***(S)-trans*-2e** and minor ***(S)-cis*-2e** isomers, 15.94.

^{1}H NMR (400 MHz, $CDCl_3$): 7.80–7.22 ppm (m, 10H), 4.89 (*trans*, q, *J* = 6.5 Hz, 1H), 4.39 (*cis*, q, *J* = 6.5 Hz, 1H), 2.71 (*trans*, m, 2H), 2.55 (*cis*, m, 2H), 1.55 (*trans*, d, *J* = 6.8 Hz, 3H), 1.45 (m, 2H), 1.37 (*cis*, d, *J* = 6.8 Hz, 3H), 0.93 (m, 3H).

(*S*)-*N*-(2-Methyl-1-phenylpropylidene)-1-phenylethanamine (2f) [Although prepared, not further examined in this study regarding its reduction]

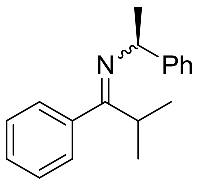

Reaction details: Use the imine-**2e** preparation method, albeit with heating (50 ^{o}C) for 24 h. *iso*-Butyrophenone, 5.00 mmol scale, 0.50 M; reaction time: 60 h; (1.08 g) 80% yield. Removal of the starting ketone required Kugelrohr distillation and provided a mixture of *cis*- and *trans*-isomers as a yellow oil. GC (program B, see General Experimental Details) retention time [min]: major **(*S*)-*cis*-2f** and minor **(*S*)-*trans*-2f** isomers, 15.00.
^{1}H NMR (400 MHz, $CDCl_3$): 7.84–6.96 ppm (m, 10H), 4.83 (*trans*, q, *J* = 6.7 Hz, 1H), 4.29 (*cis*, q, *J* = 6.5 Hz, 1H), 2.78 (m, *J* = 6.9 Hz, 1H), 1.32 (d, *J* = 6.4 Hz, 3H), 1.10 (m, 6H). See reference [S8].

CONTINUES ON THE NEXT PAGE…

Determination of trans-imines vs cis-imines. The next 20 pages are devoted to the NOESY spectra of imines 2a-e

Due to 1,3-allylic strain, the methine hydrogen (H^A), of the α-MBA moiety, is known to lie in the plane of the ketimine C–N double bond [S9-11]. As a consequence, identification of the ketimine as either *cis* or *trans* should be possible due to the proximity of proton H^A and a proton (H^B) on one of the substituents of the carbonyl carbon. The ketimine of acetophenone is shown below (See Figure 2S); a *nOe* would be expected between H^A and H^B in the *trans*-isomer only. Regarding all other substrates, the effect between the H^A and the proton H^B is clearly noticeable for the major isomer in all substrates, indicating thus their proximity in space.

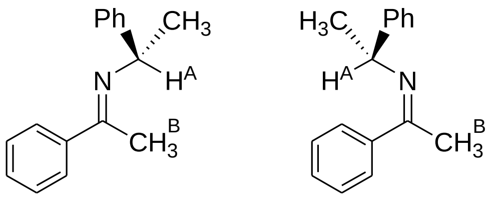

Figure S2: *nOe* is visible in the *trans*- configuration, while in the *cis*- the protons H^A and H^B are too far away.

NOTE: When considering the ^{1}H NMR spectra on the following pages, the quartet at 4.12 ppm is from the starting amine, *(S)*-PEA.

References

[1S] Hershberg, E.B. Org. Synth. 1955, Coll. Vol. 3, 626.
[2S] Maier, W. F.; Chettle, S. J.; Raghaw, S. R.; Thomas, G. *J. Am. Chem. Soc.* **1986**, *108*, 2608–2616.
[3S] Nugent, T. C.; Ghosh, A. K.; Wakchaure, V. N.; Mohanty, R. R. *Adv. Synth. Catal.* **2006**, *348*, 1289–1299.
[4S] Boyd, D.; Jennings, W. B.; Waring, L. C. *J. Org. Chem.* **1986**, *51*, 992-995.
[5S] Jennings, W. B.; Boyd, D. *J. Am. Chem. Soc.* **1972**, *94*, 7187-7188.
[6S] Overberger, C. G.; Marullo, N. P. Hiskey, R. G. *J. Am. Chem. Soc*, **1961**, *83* 1374-78.
[7S] Harada, K.; Yoshida, T. *Bull. Chem. Soc. Jpn.* **1972**, *45*(12), 3706-3710.
[8S] Arjona, O.; Perez-Ossorio, R.; Perez-Rubalcaba, A.; Plumet, J.; Santesmases, M. J. *J. Org. Chem.* **1984**, *49*, 2624-2626.
[9S] Hoffmann, R. W. *Chem. Rev.* **1998**, *89*, 1841.
[10S] Lee, K. W.; Hwang, S. Y.; Kim, C. R.; Nam, D. H.; Chang, J. H.; Choi, S. C.; Choi, B. S.; Choi, H. –W.; Lee, K. K.; So, B.; Cho, S. W.; Shin, H. *Org. Process Res. Dev.* **2003**, *7*, 839.
[11S] Nugent, T. C.; El-Shazly, M.; Wakchaure, V. *J. Org. Chem.* **2008**, *73*, 1297-1305.

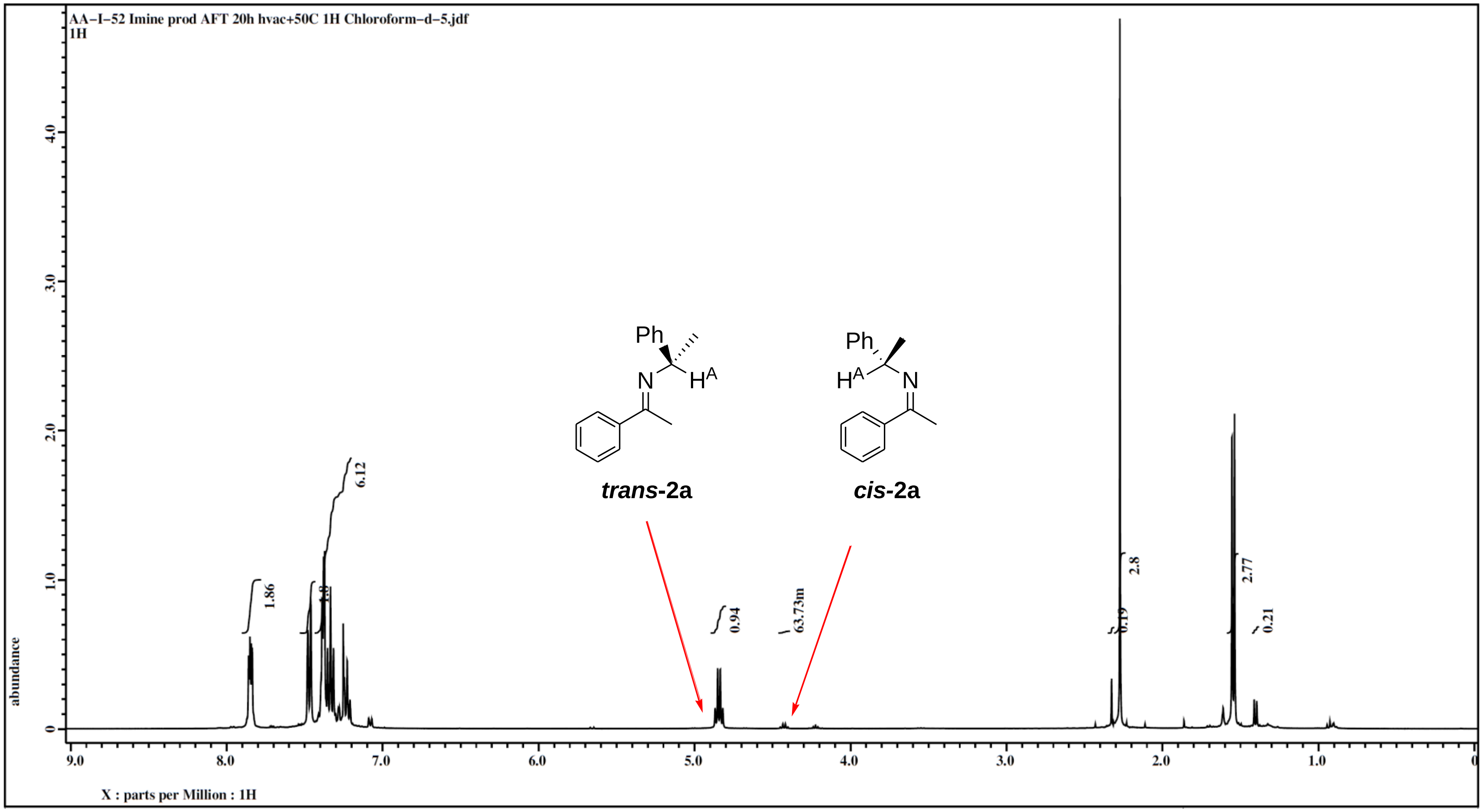

Figure S3: ^{1}H NMR spectrum of ketimine **2a**. The two quartets at 4.43 ppm and 4.85 ppm represent the methine protons (H^A) of the *cis*- and *trans*-ketimines.

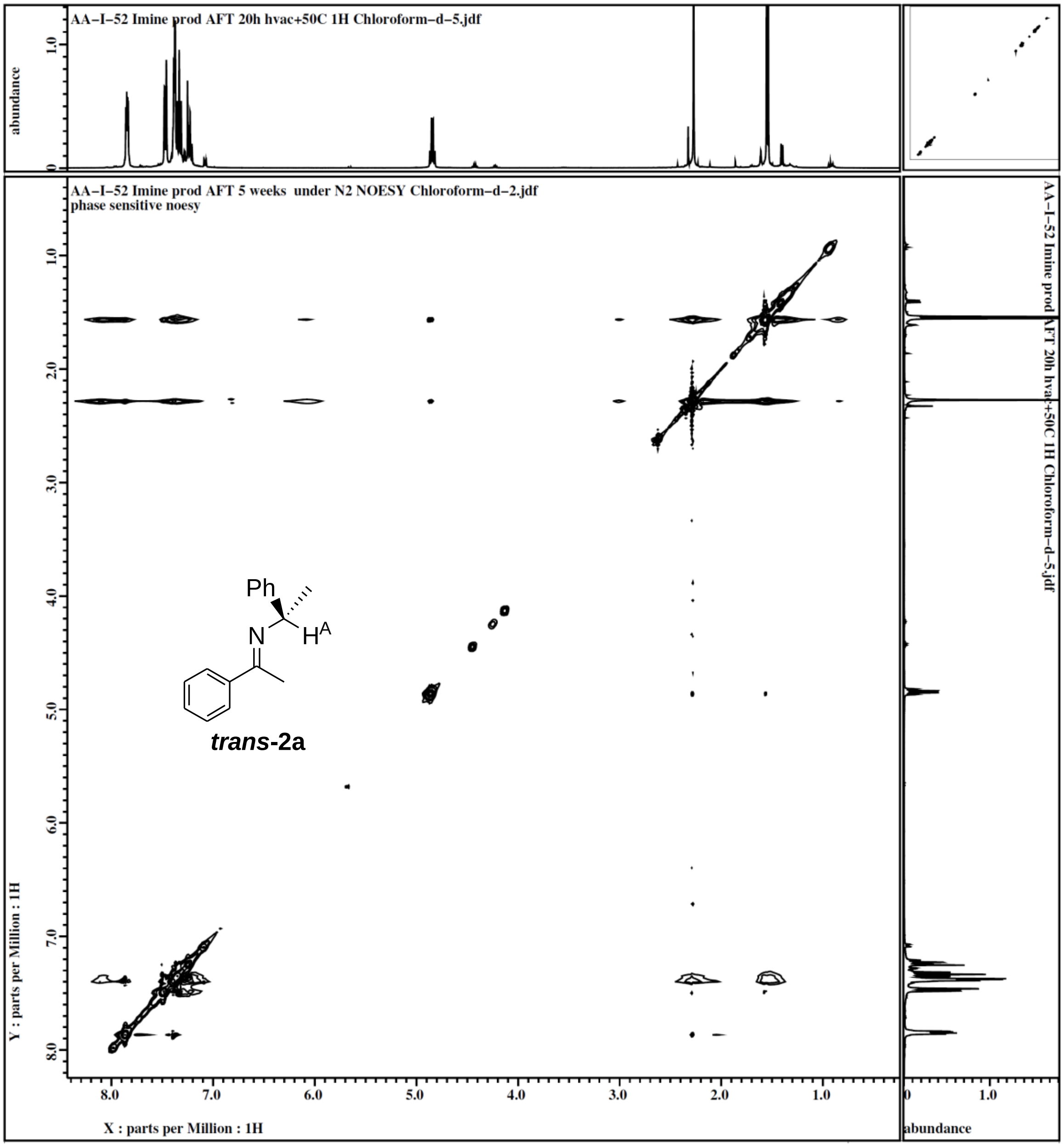

Figure S4: NOESY spectrum of ketimine **2a**.

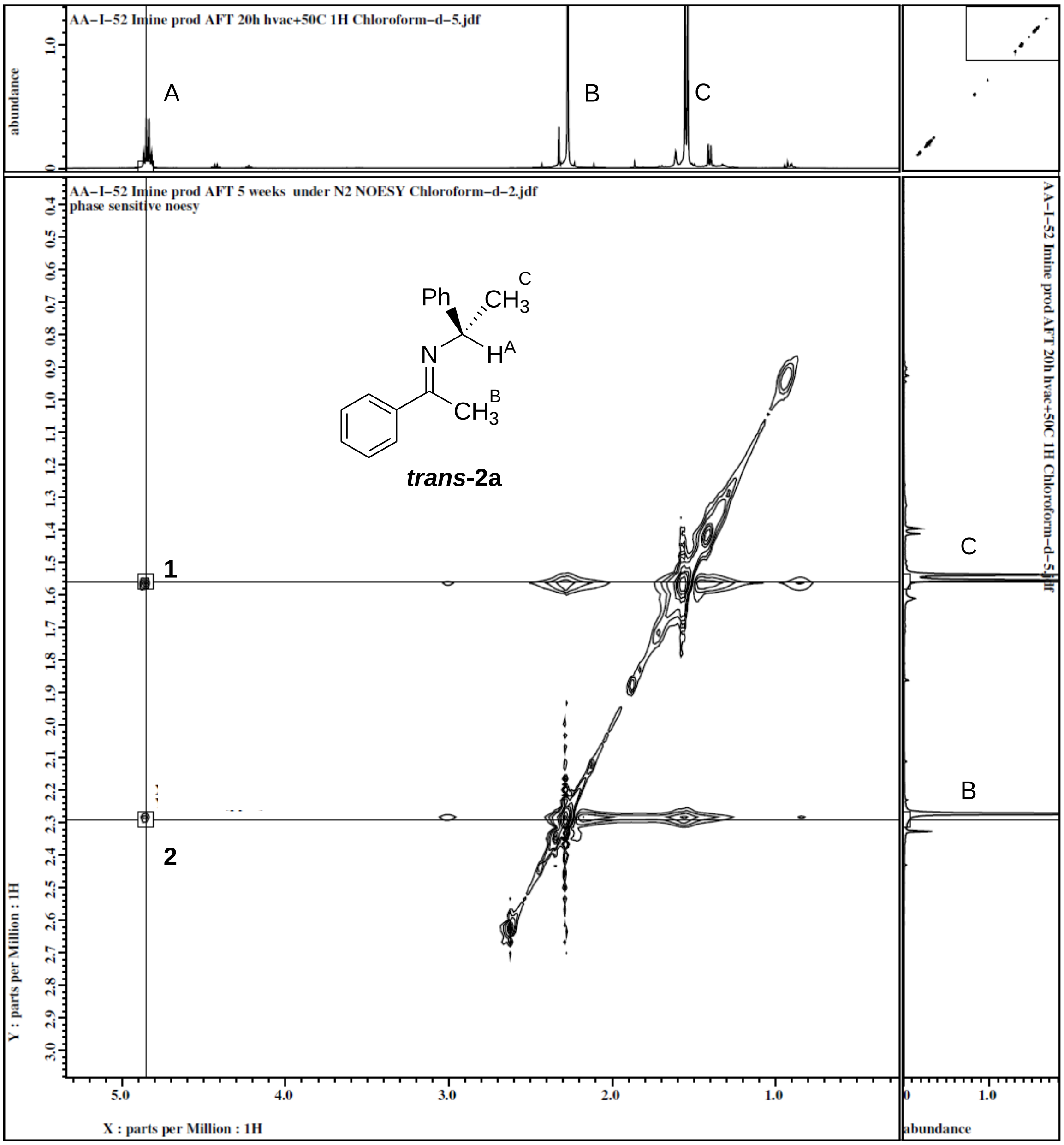

Figure S5: Expansion from Fig.4S (y-axis 0.4–3.0 ppm; x-axis 0.3–5.3 ppm).
Cross Peak labeled 1. The major quartet at 4.85 ppm (A) correlates with the major doublet of the methyl at 1.53 ppm (C).
Cross Peak labeled 2. The quartet at 4.85 ppm (A) also correlates with the major singlet at 2.27 ppm (B).
The data strongly suggests that the major ketimine diastereomer is *trans*-**2a**.

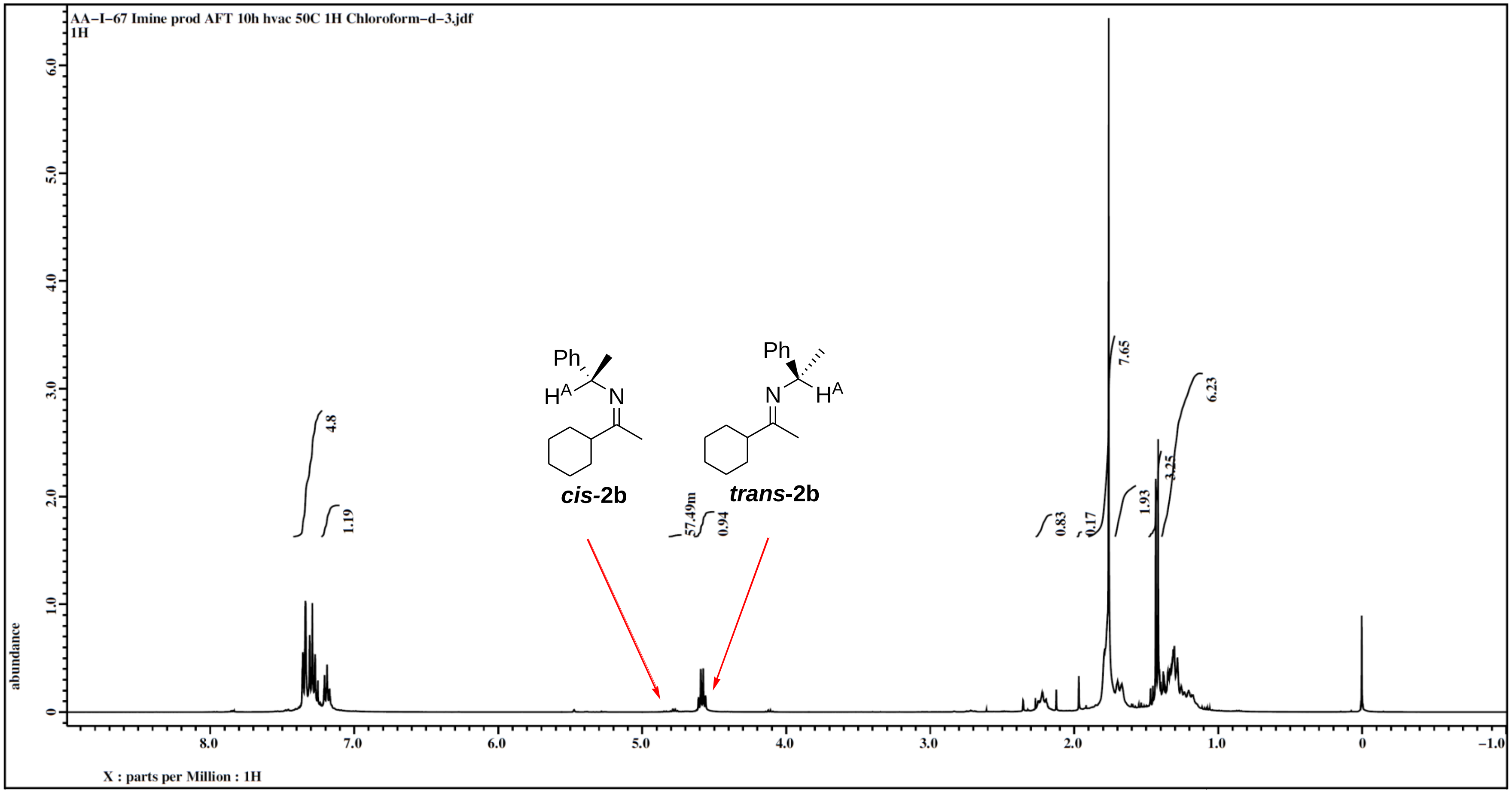

Figure S6: ^{1}H NMR spectrum of ketimine **2b**. The two quartets at 4.57 ppm and 4.75 ppm represent the methine protons (H^A) of the *cis*- and *trans*-ketimines.

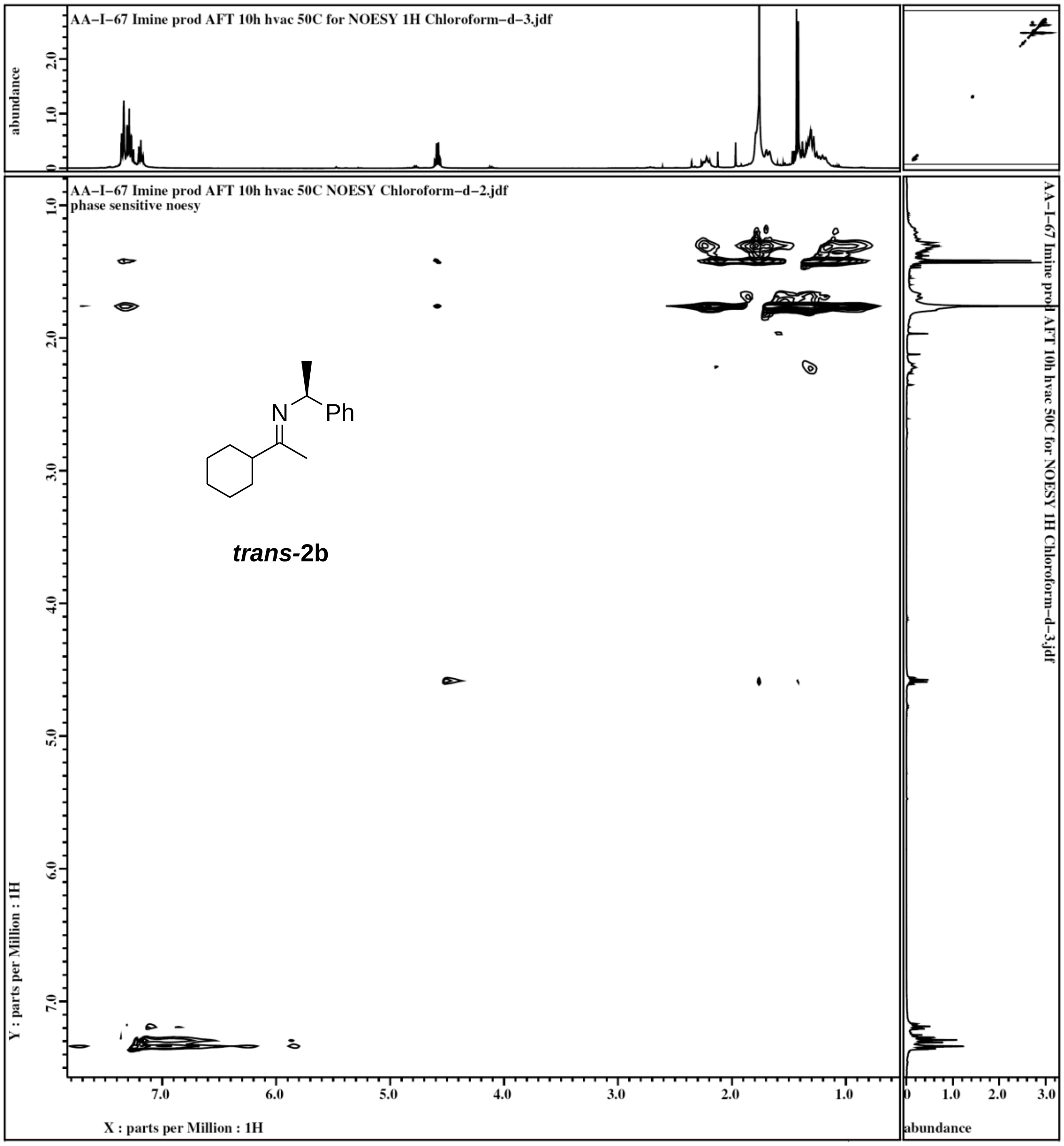

Figure S7: NOESY spectrum of ketimine **2b**.

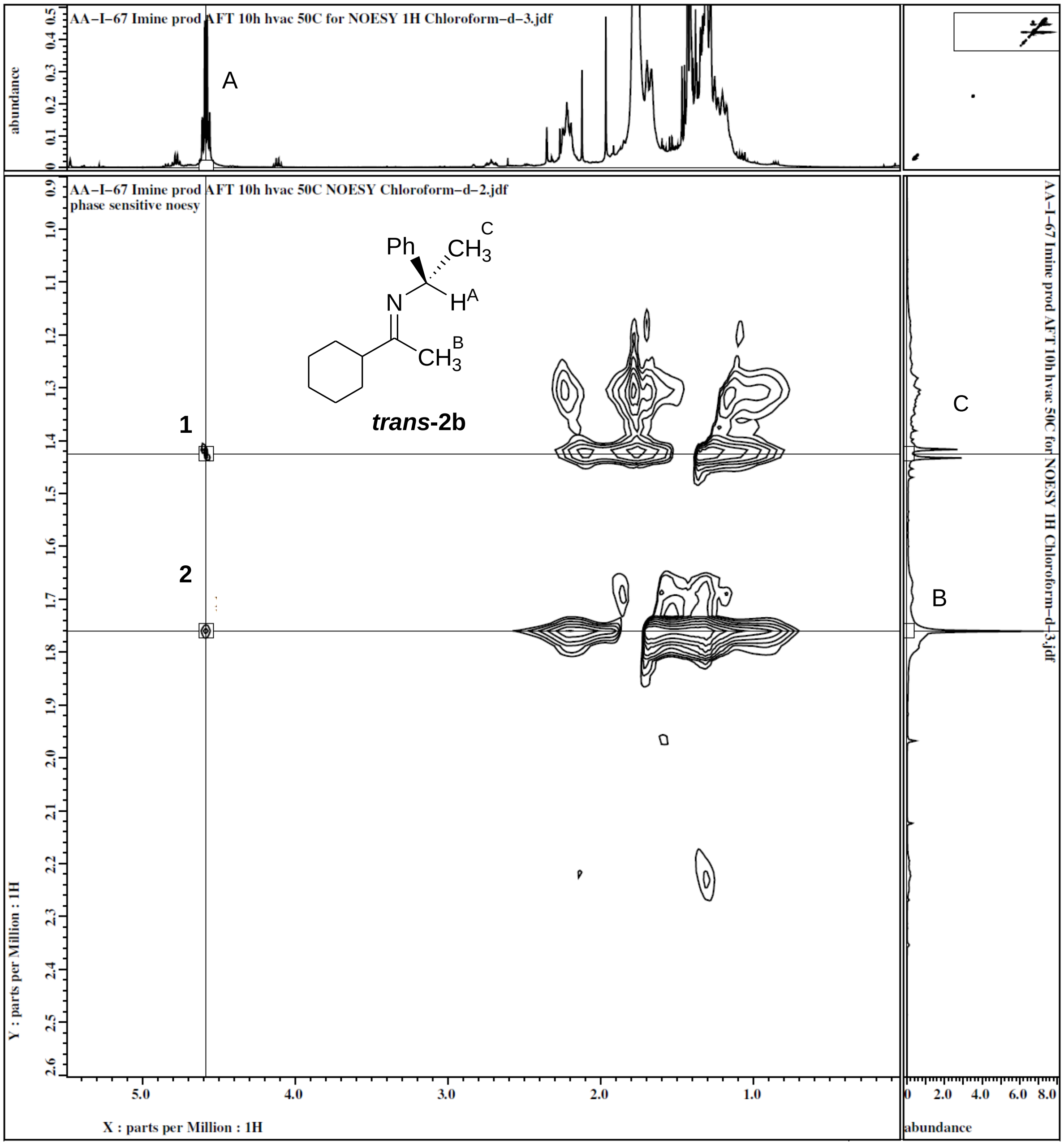

Figure S8: Expansion from Fig.7S (y-axis 0.9–2.6 ppm; x-axis 0.0– 5.4 ppm).
Cross Peak labeled 1.. The major quartet at 4.57 ppm (A) correlates with the doublet of the methyl at 1.43 ppm (C).
Cross Peak labeled 2.. At the same time, it correlates with the singlet at 1.76 ppm (B), clearly suggesting that the major diastereomer is *trans*-**2b**.

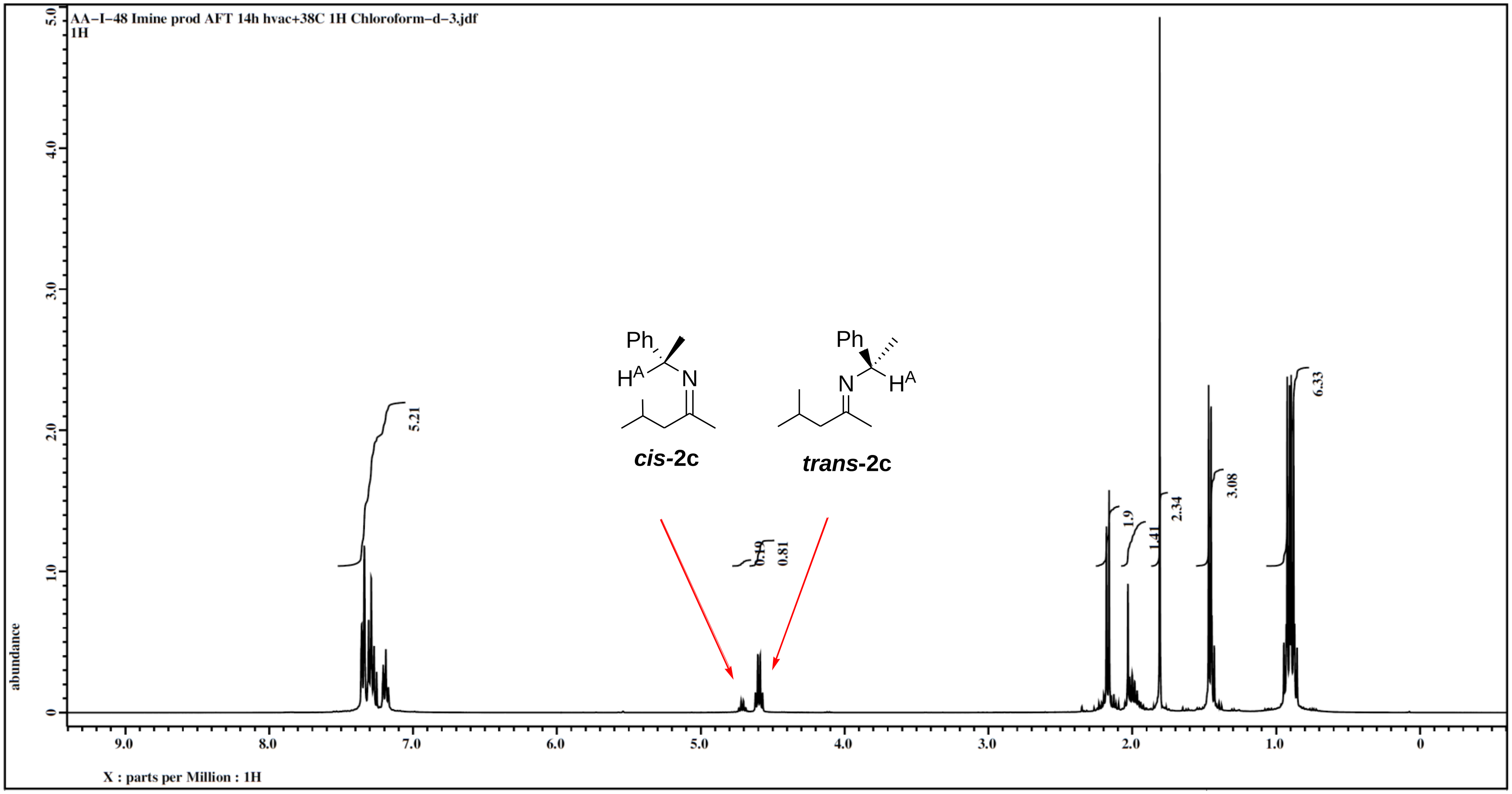

Figure S9: ^{1}H NMR spectrum of ketimine **2c**. The two quartets at 4.58 ppm and 4.62 ppm represent the benzylic methine proton (H^{A}) of the ketimine.

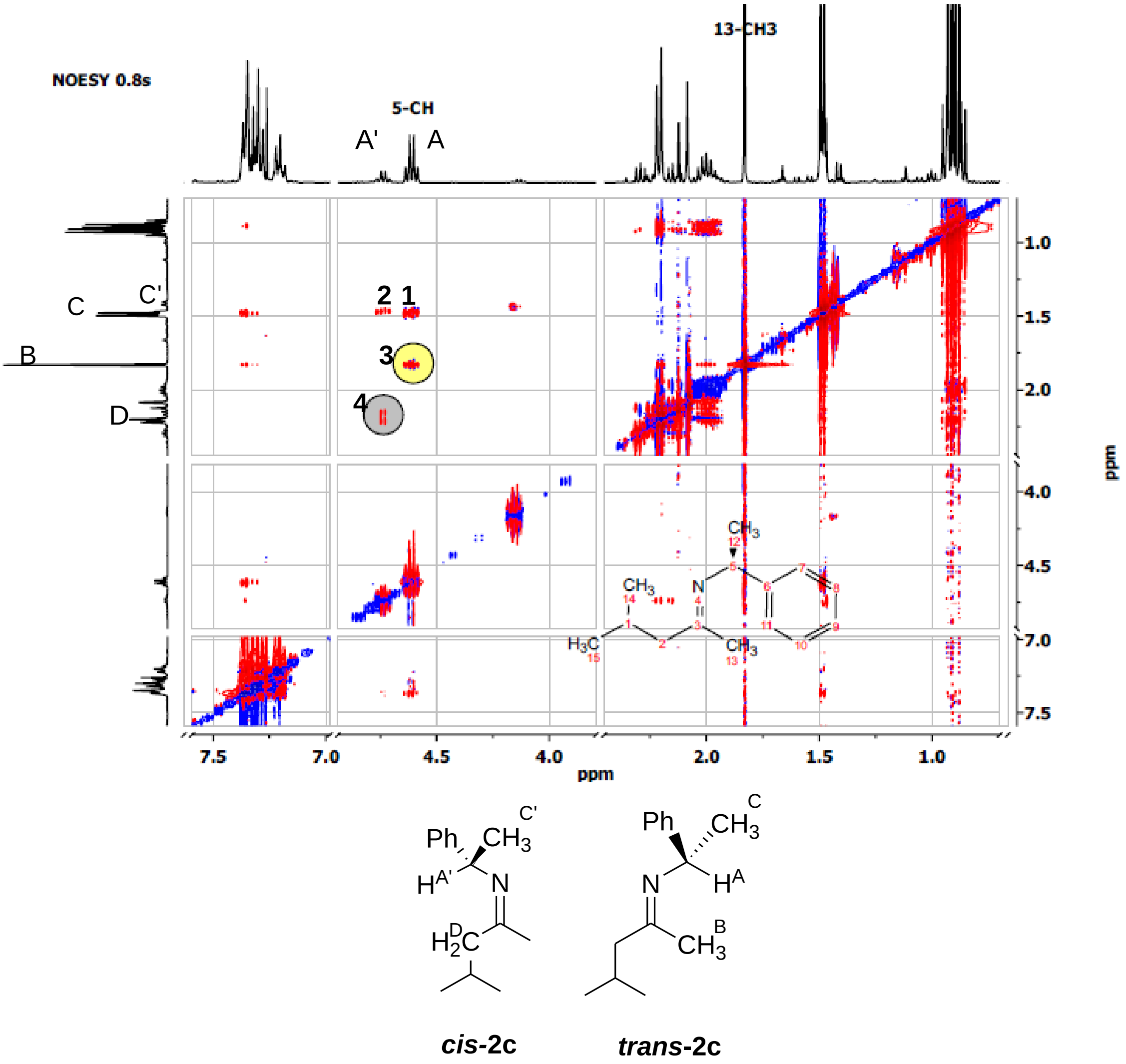

Figure S10: NOESY spectrum of ketimine **2c**.

Cross peak labeled at 1. The major quartet at 4.58 ppm (A) correlates with the doublet of the methyl at 1.47 ppm (C).

Cross peak labeled at 2. The same correlation can be seen for the minor quartet (A') with the minor doublet for the resonance at 1.44 ppm (C').

Cross peak labeled at 3. The major quartet (A) correlates with the singlet at 1.81 ppm (B).

Cross peak labeled at 4. The minor quartet (A') correlates with the methylene protons at 2.18 ppm (D)

This combination of data strongly suggests that the major diastereomer is *trans*-**2c** and simultaneously confirms the minor ketimine as ***cis*-2c**.

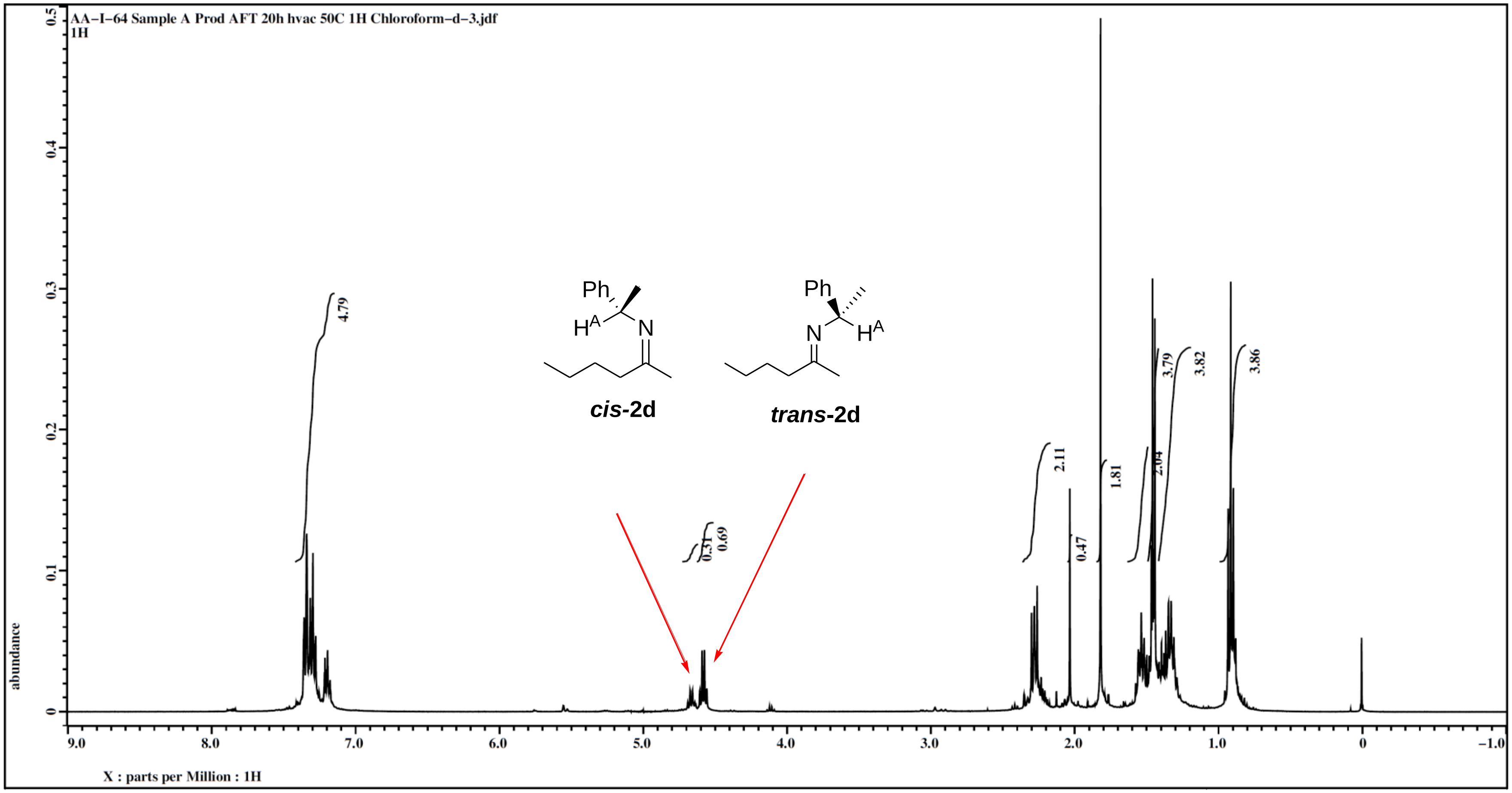

Figure S11: ^{1}H NMR spectrum of ketimine **2d**. The two quartets at 4.59 ppm and 4.55 ppm represent the methine proton (H^A) of the ketimine.

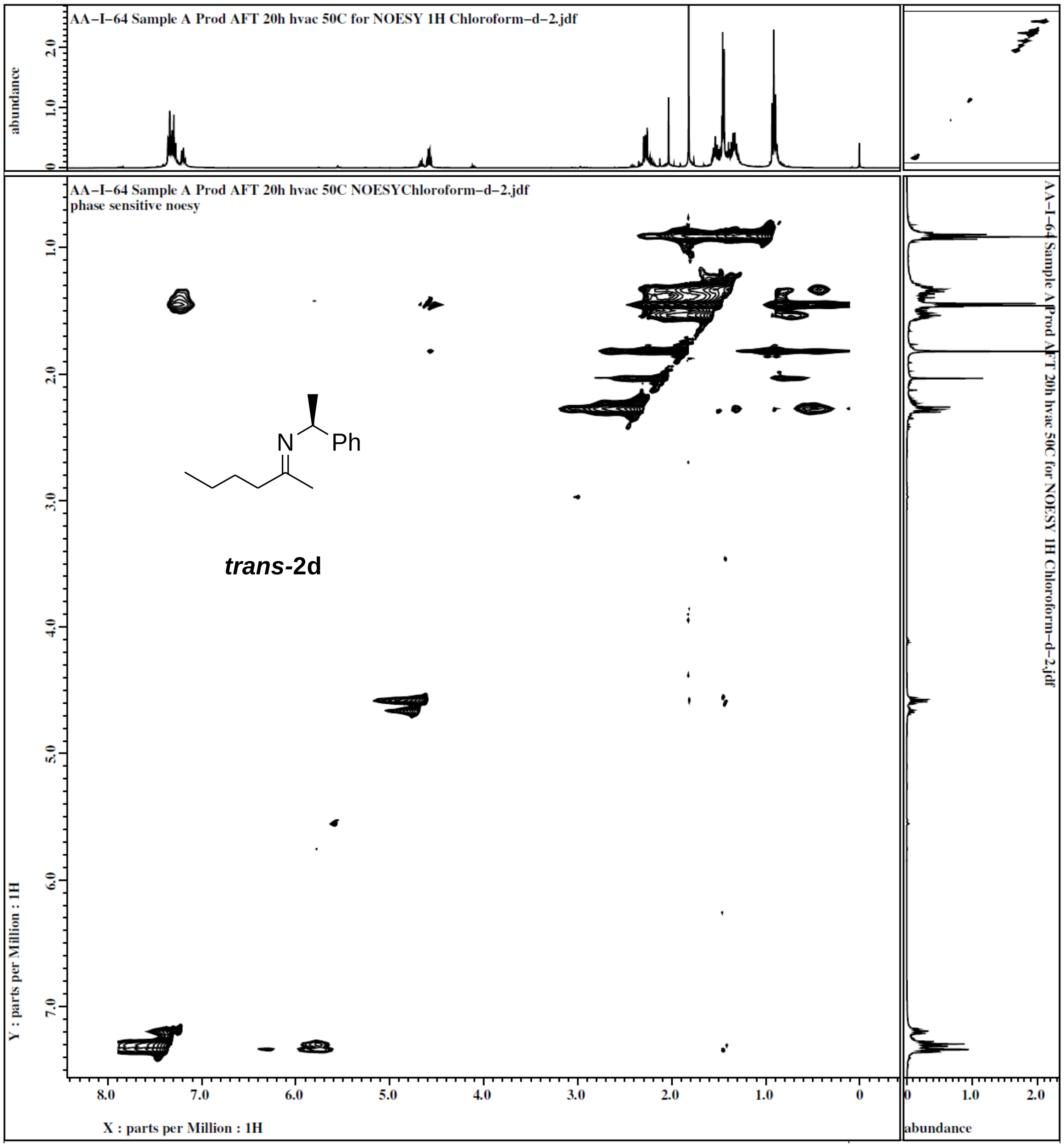

Figure S12: NOESY spectrum of ketimine **2d**.

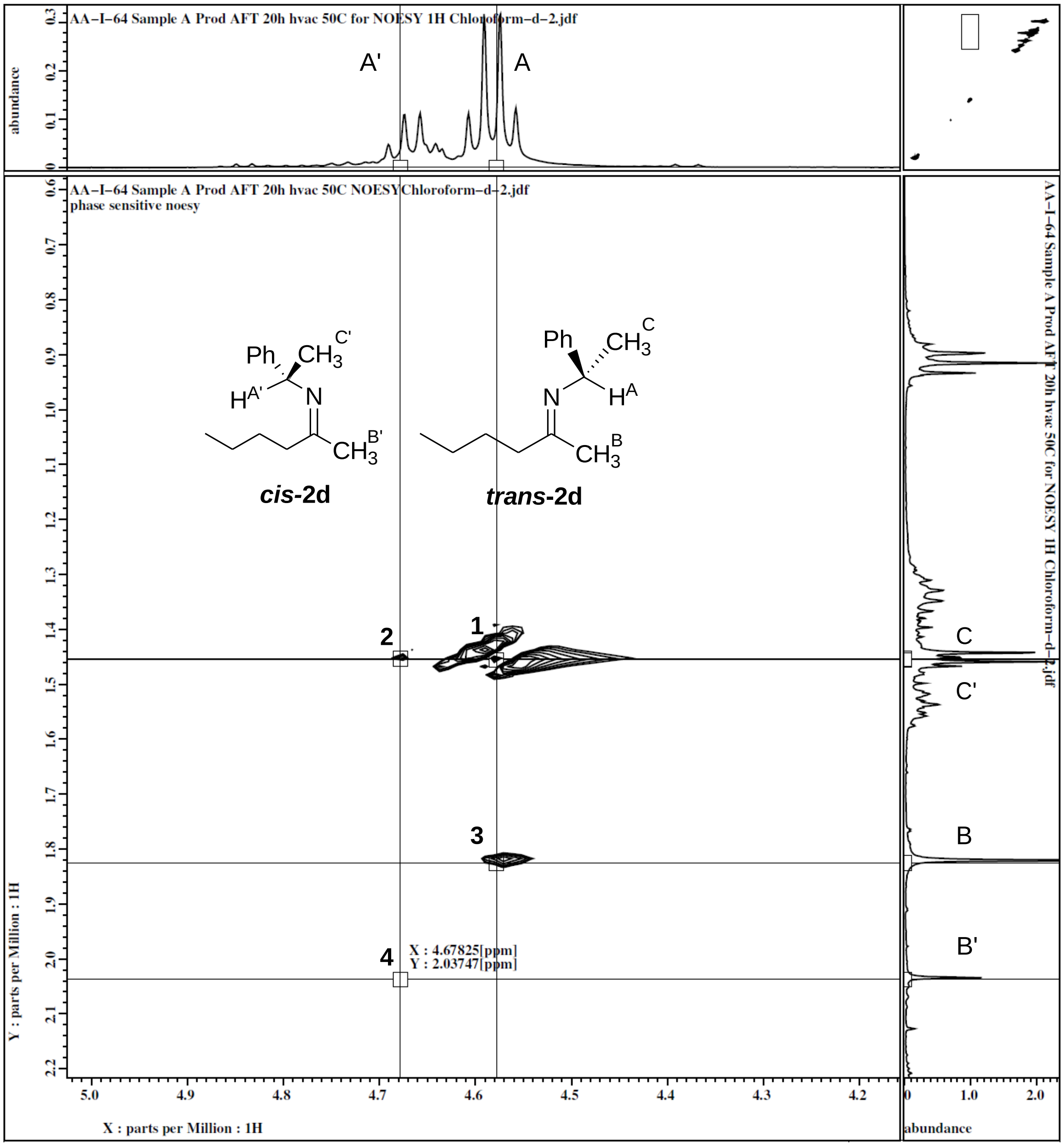

Figure S13: Expansion from Fig.12S (y-axis 0.6– 2.2 ppm; x-axis 4.16– 5.2 ppm).
Cross Peak labeled 1. The major quartet at 4.55 ppm (A) correlates with the doublet of the methyl at 1.44 ppm (C).
Cross Peak labeled 2. The same correlation can be seen for the minor quartet (A'), although the doublet for the minor resonates at δ 1.45 (C').
Cross Peak labeled 3. The major quartet (A) also correlates with the singlet at δ 1.82 (B) clearly suggesting that the major diastereomer is *trans*-**2d.**
Cross Peak labeled 4. Correlation of the minor singlet at δ 2.03 (B') and the minor quartet (A') cannot be seen; this confirms the minor diastereomer to be the *cis*-**2d**.

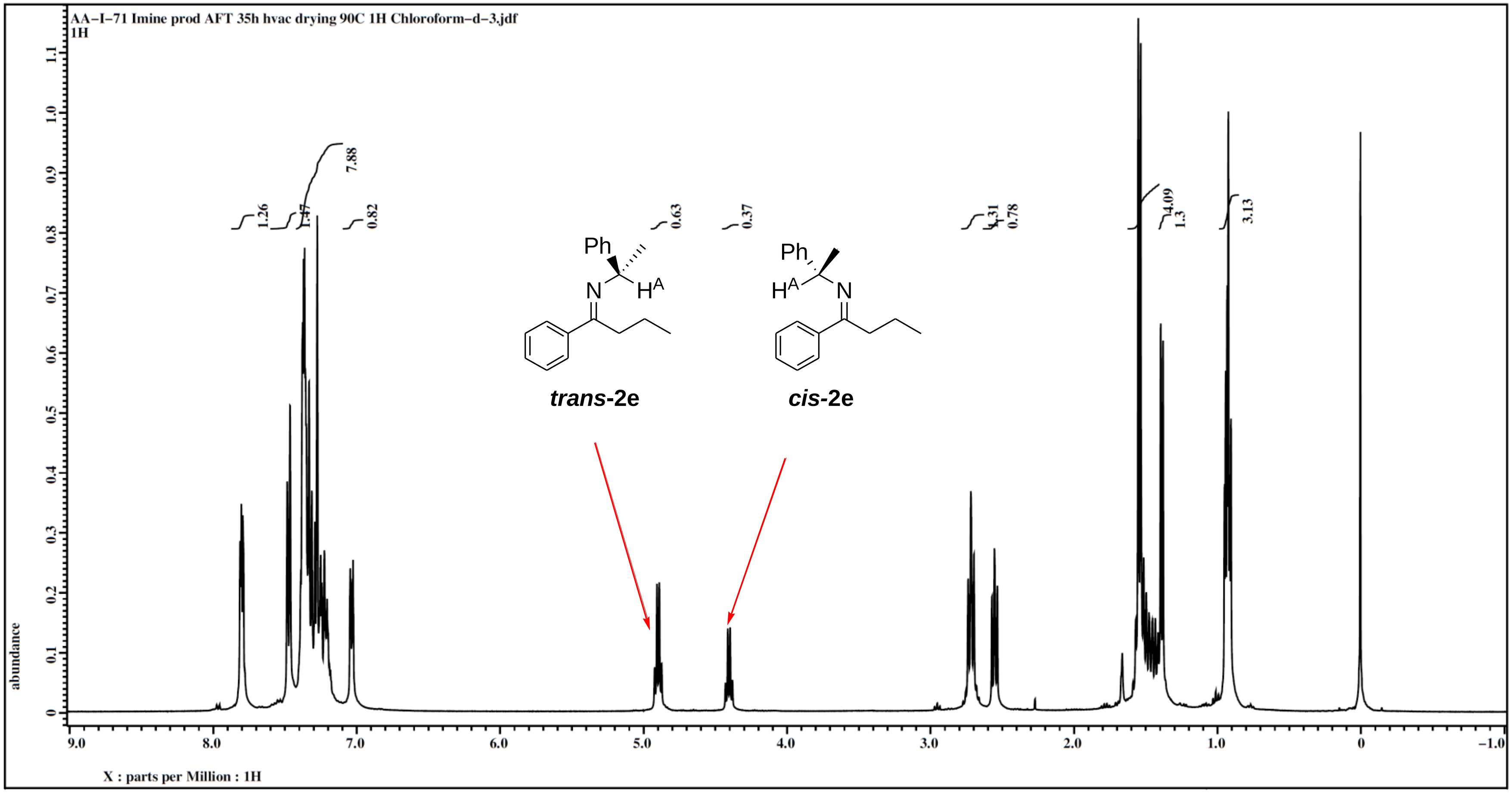

Figure S14: ^{1}H NMR spectrum of ketimine **2e**. The two quartets at 4.89 ppm and 4.39 ppm represent the methine proton (H^{A}) of the ketimine.

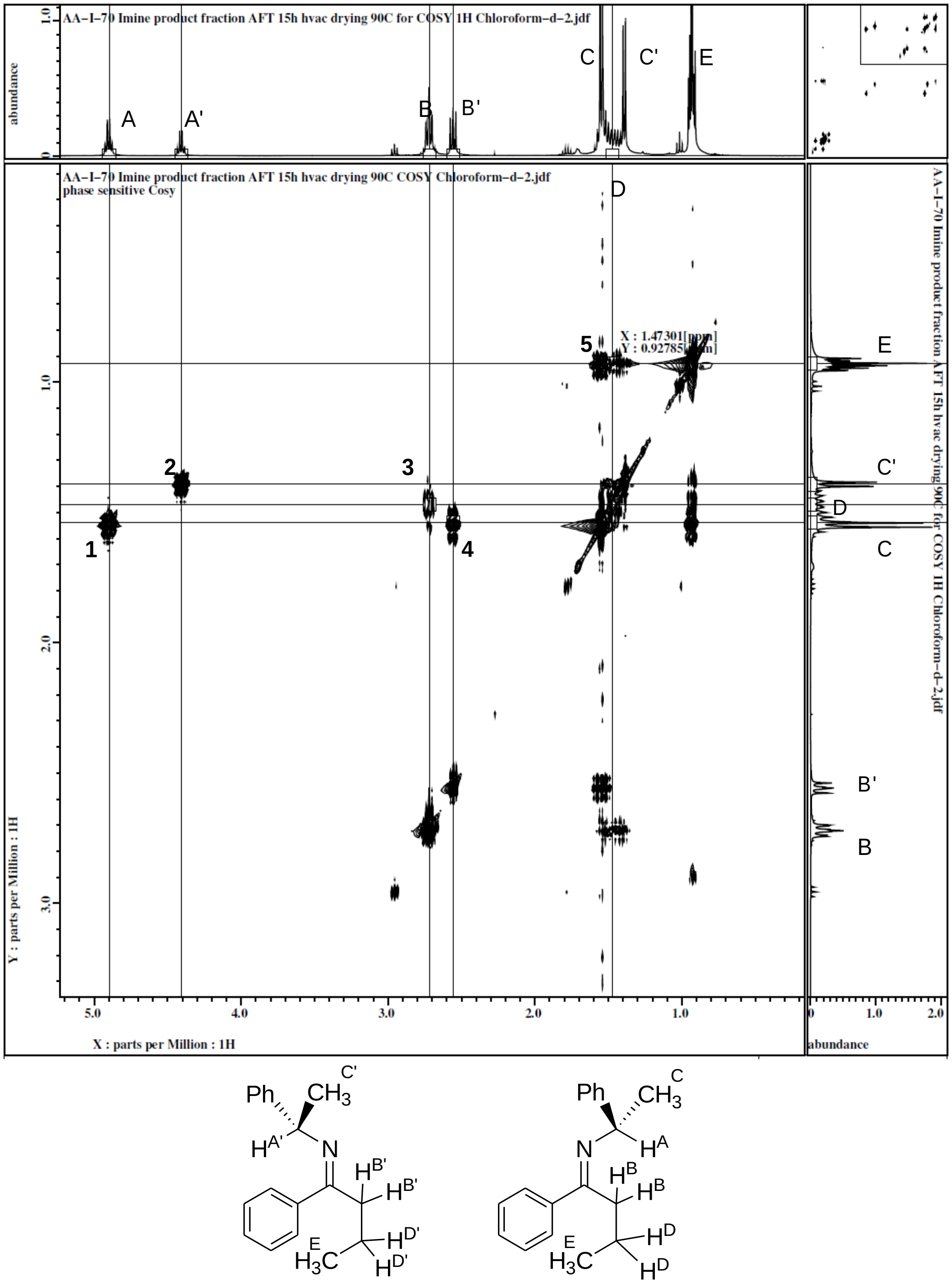

Figure S15: Expansion of COSY spectrum of ketimine **2e** (y-axis 0.2–3.6 ppm; x-axis 0.2– 5.2 ppm).

Cross Peak labeled 1. The major quartet at 4.89 ppm (A) correlates with the major doublet of the methyl at 1.53 ppm (C). **Cross Peak labeled 2.**. The same correlation can be seen for the minor quartet (A’), although the doublet for the minor resonates at 1.39 ppm (C’).

Cross Peak labeled 3. The major multiplet (B) correlates with a buried multiplet at 1.45 ppm (D). **Cross Peak labeled 4.** The minor multiplet (B’) also correlates with the buried multiplet, in this case D’. **Cross Peak labeled 5.** The buried multiplet was assigned from the interaction of the terminal methyl protons (E).

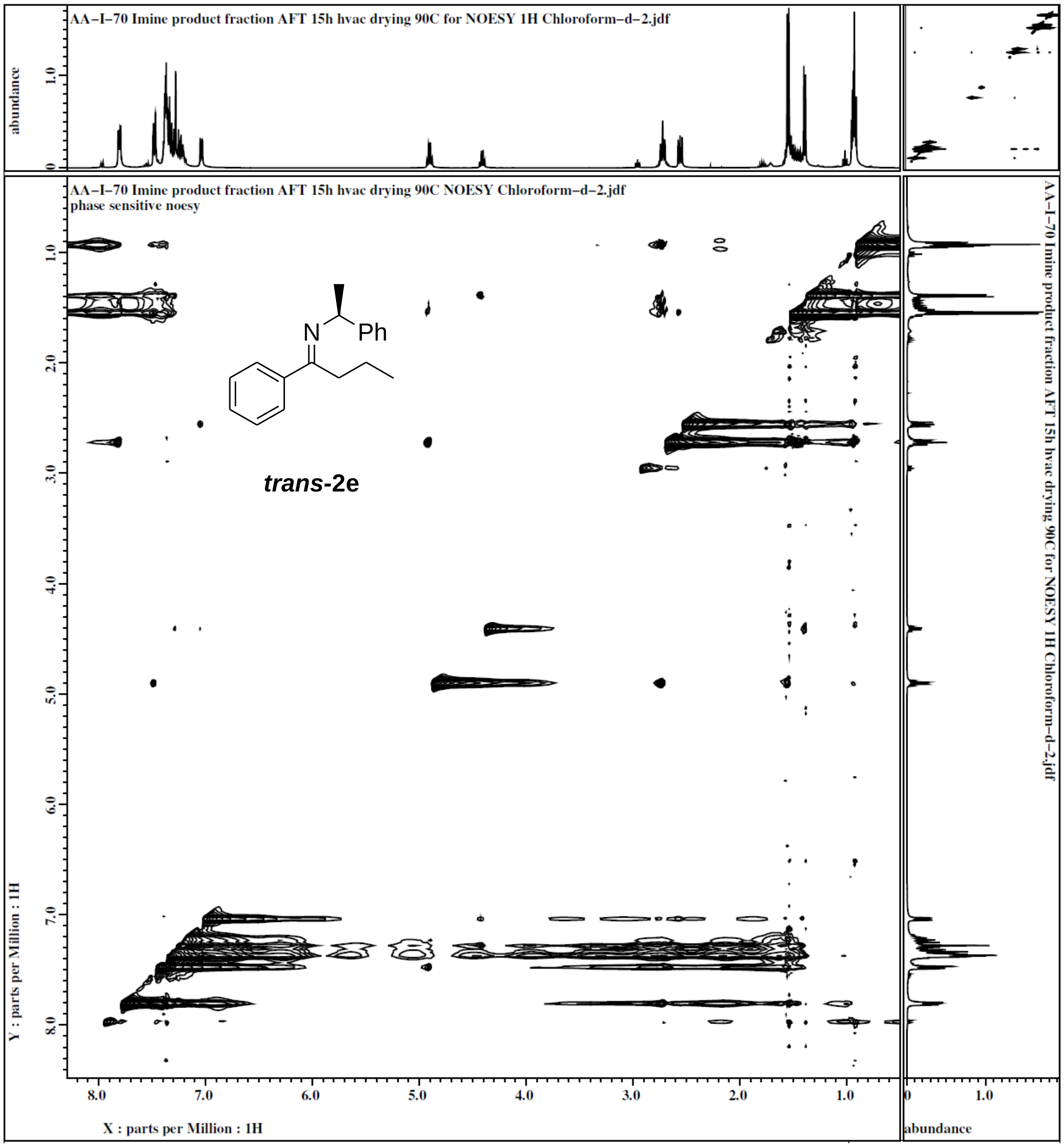

Figure S16: NOESY spectrum of ketimine **2e**.

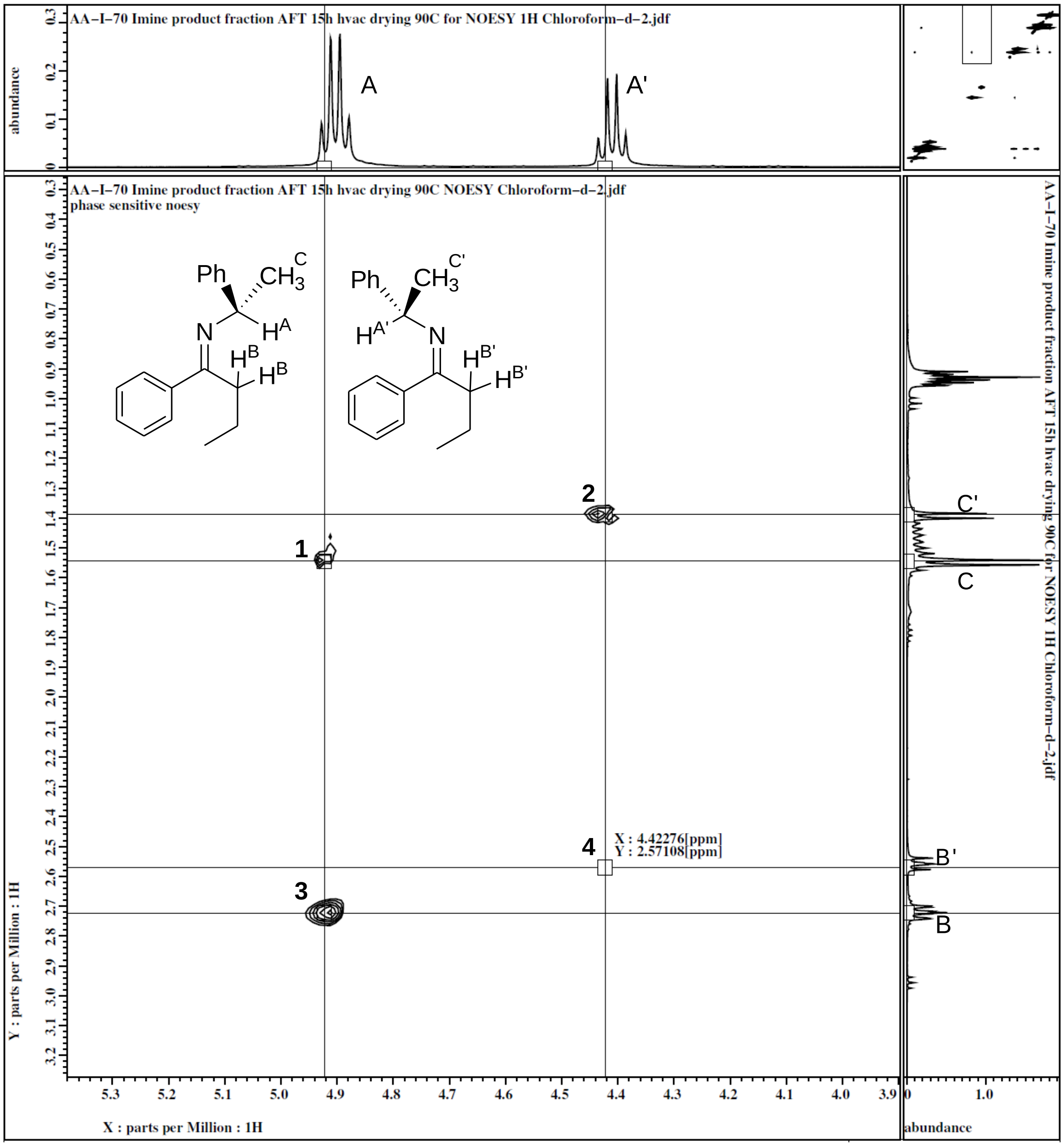

Figure S17: Expansion from Fig.16S (y-axis 0.28–3.26 ppm; x-axis 3.90–5.36 ppm).
Cross Peak labeled 1. The major quartet at 4.89 ppm (A) correlates with the major doublet of the methyl at 1.55 ppm (C).
Cross Peak labeled 2. The same correlation can be seen for the minor quartet (A'), although the doublet for the minor resonates at 1.37 ppm (C').
Cross Peak labeled 3. The major quartet (A) also correlates with a major multiplet at 2.71 ppm (B) clearly suggesting that the major diastereomer is *trans*-**2e**.
Cross Peak labeled 4. Correlation of the minor multiplet at 2.55 ppm (B') and the minor quartet (A') cannot be seen; this confirms the minor diastereomer to be the ***cis*-2e**.

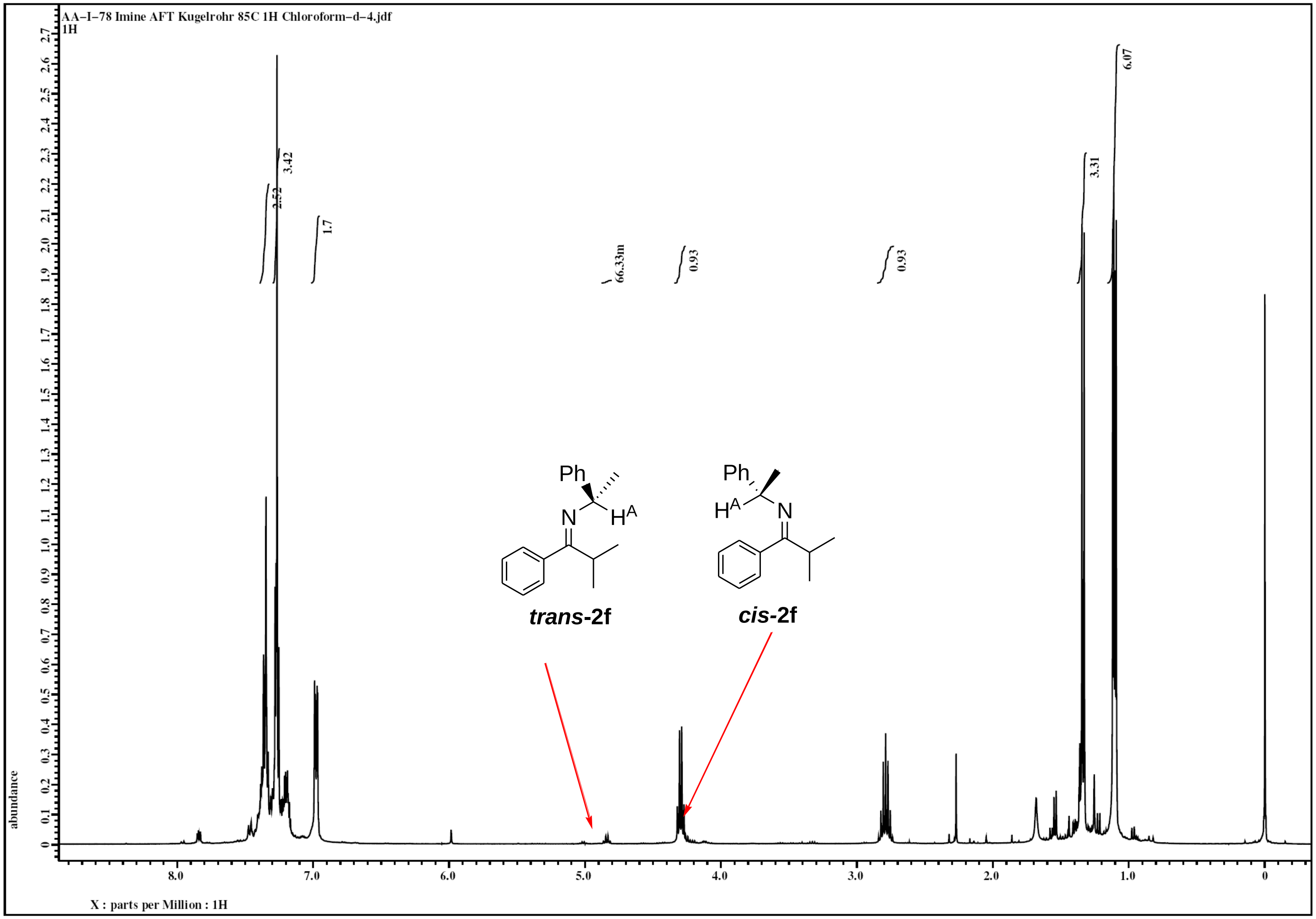

Figure S18: ^{1}H NMR spectrum of ketimine **2f** (not further examined in this study). The two quartets at 4.29 ppm and 4.83 ppm represent the methine proton (H^A) of the ketimine.

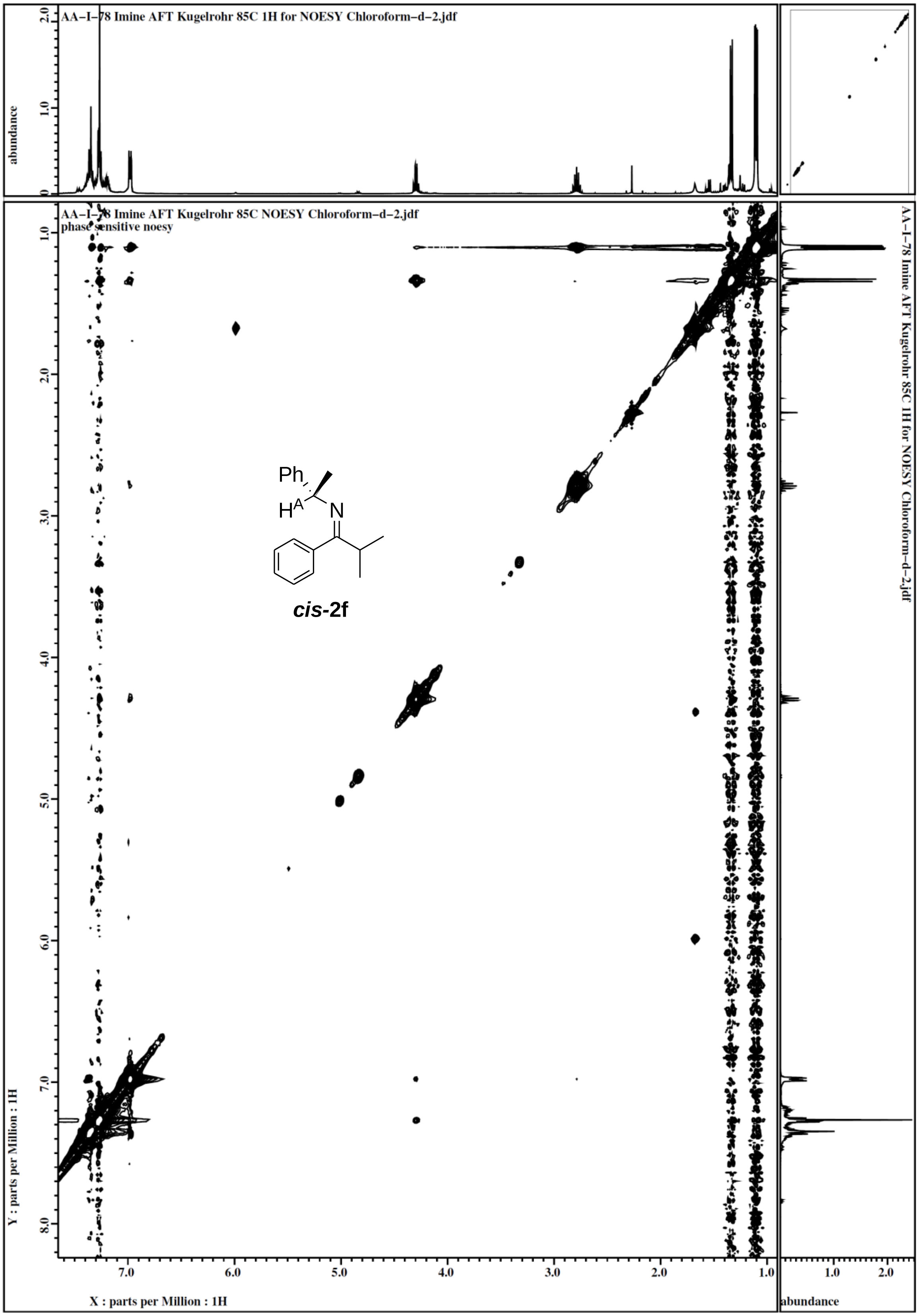

Figure S19: NOESY spectrum of ketimine **2f**.

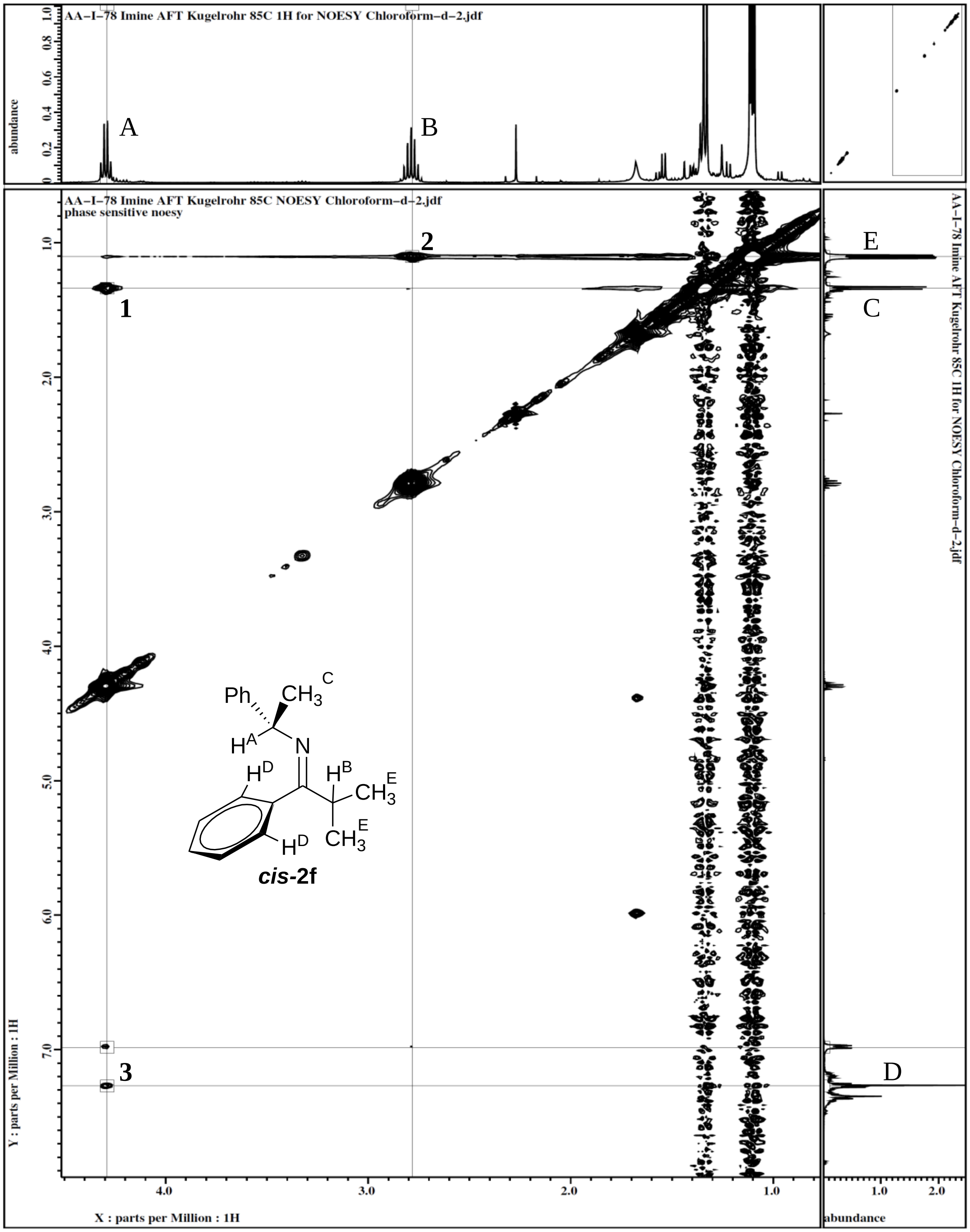

Figure S20: Expansion from Fig.19S (y-axis 0.6– 7.9 ppm; x-axis 0.78–4.51 ppm).
Cross Peak labeled 1. The major quartet at 4.29 ppm (A) correlates with the doublet of the methyl at 1.32 ppm (C).
Cross Peak labeled 2. Multiplet at 2.78 ppm (B) correlates with the multiplet at 1.10 ppm (E).
Cross Peak labeled 3. The major quartet (A) also correlates with resonances in the aromatic area (D), clearly suggesting that the major diastereomer is *cis*-**2f**.
